# The SYNBREED chicken diversity panel: a global resource to assess chicken diversity at high genomic resolution

**DOI:** 10.1186/s12864-019-5727-9

**Published:** 2019-05-07

**Authors:** Dorcus Kholofelo Malomane, Henner Simianer, Annett Weigend, Christian Reimer, Armin Otto Schmitt, Steffen Weigend

**Affiliations:** 1 Animal Breeding and Genetics Group, Department of Animal Sciences, University of Goettingen, 37075 Goettingen, Germany; 2 Center for Integrated Breeding Research, Department of Animal Sciences, University of Goettingen, 37075 Goettingen, Germany; 3 Breeding Informatics Group, Department of Animal Sciences, University of Göttingen, 37075 Göttingen, Germany; 4 Institute of Farm Animal Genetics, Friedrich-Loeffler-Institut, 31535 Neustadt, Germany

**Keywords:** SYNBREED panel, Global chickens, Genetic diversity, Fancy breeds, SNPs

## Abstract

**Background:**

Since domestication, chickens did not only disperse into the different parts of the world but they have also undergone significant genomic changes in this process. Many breeds, strains or lines have been formed and those represent the diversity of the species. However, other than the natural evolutionary forces, management practices (including those that threaten the persistence of genetic diversity) following domestication have shaped the genetic make-up of and diversity between today’s chicken breeds. As part of the SYNBREED project, samples from a wide variety of chicken populations have been collected across the globe and were genotyped with a high density SNP array. The panel consists of the wild type, commercial layers and broilers, indigenous village/local type and fancy chicken breeds. The SYNBREED chicken diversity panel (SCDP) is made available to serve as a public basis to study the genetic structure of chicken diversity. In the current study we analyzed the genetic diversity between and within the populations in the SCDP, which is important for making informed decisions for effective management of farm animal genetic resources.

**Results:**

Many of the fancy breeds cover a wide spectrum and clustered with other breeds of similar supposed origin as shown by the phylogenetic tree and principal component analysis. However, the fancy breeds as well as the highly selected commercial layer lines have reduced genetic diversity within the population, with the average observed heterozygosity estimates lower than 0.205 across their breeds’ categories and the average proportion of polymorphic loci lower than 0.680. We show that there is still a lot of genetic diversity preserved within the wild and less selected African, South American and some local Asian and European breeds with the average observed heterozygosity greater than 0.225 and the average proportion of polymorphic loci larger than 0.720 within their breeds’ categories.

**Conclusions:**

It is important that such highly diverse breeds are maintained for the sustainability and flexibility of future chicken breeding. This diversity panel provides opportunities for exploitation for further chicken molecular genetic studies. With the possibility to further expand, it constitutes a very useful community resource for chicken genetic diversity research.

**Electronic supplementary material:**

The online version of this article (10.1186/s12864-019-5727-9) contains supplementary material, which is available to authorized users.

## Background

Chickens are of major and increasing importance for agricultural production as an efficient source of high quality protein. There have been concerns about loss of animal genetic resources and erosion of many genotypes due to crossbreeding or replacement by the high performing commercial hybrids resulting from highly efficient selection programs [1, 2]. Such loss of valuable genetic material will put a strain on animal production and could make it vulnerable to challenges in the future. It is therefore important to preserve genetic resources that may help to meet future demands in animal breeding [3, 4]. Studying and understanding the diversity between and within populations clearly is crucial for effective management of farm animal genetic resources [5].

Domestication history of chickens is still a matter of scientific debate, and has enjoyed the interest of researchers and scholars, from tracing the centers of domestication to exploring the archeology and dispersion of the chickens across different parts of the world [6–10]. One widely accepted hypothesis is that the main source of today’s chickens which are diffused across the world comes from domestication events that have taken place in the Indus Valley during 2500–2100 B.C. [6, 11]. Since domestication, chickens have been widely dispersed from Asia to the different parts of the world. Several routes from the centers of domestication to Europe, Africa and South America have been reported [9, 10, 12–14]. From Asia, chickens are believed to have reached Europe through the Mediterranean region and through the north via China and Russia to Northern Europe [6]. It is supposed that chickens in Africa have descended from both European and Asian chicken stocks [6, 8]. Despite the debate on whether the South American chickens originated from Polynesian or European breeds [9, 13, 15, 16], it is clear that both European and Asian flocks have contributed to the South American chicken breeds. Several local Asian and European breeds have formed the founder stocks to develop commercial egg laying and broiler chickens. Subsequently, the commercial lines have been highly selected for production purposes (e.g. meat, egg production and feed conversion efficiency) [5, 12, 17].

In Europe, many local type breeds were developed mostly by intense selection and crossbreeding for desired phenotypic traits. In the nineteenth century, with an increasing popularity local strains maintained for centuries in Europe have been developed into standardized chicken breeds. At the same time, Asian breeds such as Cochin and Langshan were imported to Europe. In addition to keeping them as purebred populations, many new breeds evolved from crossing European breeds and newly imported Asian breeds following the European Poultry Standards [18]. Fancy chicken breeding in Europe is characterized by limited exchange of mating individuals resulting in population fragmentation, which promotes inbreeding when population sizes are small. In Asian, African and South American countries, however, local chicken breeds are often raised by villagers under extensive farming systems and with little to no selection, and exchange of breeding stocks across close villages [1, 19–23]. Due to the often low productivity of local, unselected breeds in many developing countries, the production of local breeds has been threatened by the commercial breeding and the introduction of crossbreeding to improve productivity [8, 20, 24].

The history of the origin of chickens together with management practices following domestication provides an important backbone to assess the genetic make-up and diversity between today’s chicken breeds. Low resolution studies of chicken biodiversity using microsatellites have shown that genetic diversity has been greatly affected by management practices. Highly selected layer lines, in particular white layers, showed reduced genetic variability while the wild type and less improved indigenous village chickens retained high genetic diversity [25, 26]. In this study we used single nucleotide polymorphism (SNP) genotype data to study the biodiversity of a wide range of globally sampled chicken populations at a high genomic resolution. This data was acquired under the umbrella of the SYNBREED (www.synbreed.tum.de) project. The SYNBREED chicken diversity panel used here consists of 174 chicken populations, representing four continents (Asia, Europe, South America and Africa). The SCDP also includes broiler and layer purebred lines, as well as two wild populations (*Gallus gallus gallus* and *Gallus gallus spadiceus*). We have included some commercial lines in our analyses as representatives of the most favored stocks in breeding programs whose end products are distributed globally. They are not at risk for extinction, but may threaten local breeds by crossbreeding. We show their share of genetic diversity with a much wider spectrum of chicken breeds in SCDP set which these commercial lines do not cover. We have analyzed the genetic diversity within and between the populations and report here the current status of global genetic diversity based on this panel.

## Methods

### Data acquisition

#### Animals

Deoxyribonucleic acid (DNA) samples were collected from a wide range of chicken populations across the globe under the umbrella of the SYNBREED project (project lifetime 2009–2014). First, samples were collected from 80 fancy chicken breeds in Germany between 2010 and 2012. Fancy breeds are chickens which have been developed following hobbyists’ selection programs to create phenotypes which meet the requirements of the poultry standards (i.e. the European Poultry Standards). The German Association of Poultry Breeders (Bund Deutscher Rassgeflügelzüchter e.V., BDRG) maintains a wide spectrum of traditional and fancy poultry breeds. They reflect various types of breeds of very different origins and breed histories according to the European Poultry Standards. Additional samples were collected from chicken breeds kept by farmers organized in “The Society for the Conservation of Old and Endangered Livestock Breeds (Gesellschaft zur Erhaltung alter und gefährdeter Haustierrassen e.V., GEH)”. Blood samples were collected from the wing vein using EDTA as anticoagulant. Sampling was carried out in strict accordance to the German Animal Welfare regulations, and notice was given to the authorities of Lower Saxony according to § 8 of the German Animal Welfare Act (33.9–42,502-05-10A064) and with the written consent of the animal owners. The collection was completed by samples of two Red Jungle Fowl populations, *Gallus gallus gallus* and *Gallus gallus spadiceus*, as well as samples of nine local breeds and four broiler lines taken from the previous EU project AVIANDIV (https://aviandiv.tzv.fal.de/, see also [25]). In addition, four commercial purebred white layer lines and four commercial purebred brown layer lines were added from other subprojects of the SYNBREED project.

After 2012, the panel was complemented with DNA samples of 71 populations from 22 countries provided by partners (see Table 1) or taken from previous collaborations. The total data used in this study consisted of 3235 individuals from 162 populations (from 32 countries, representing the Africa, South America, Asia, and Europe) and 12 commercial purebred lines (4 white egg layers, 4 brown egg layers and 4 broilers). The breeds’ information (i.e. names, acronyms, samples sizes and other information) is presented in Additional file 1: Table S1. The populations labeled (in the ‘Label’ column) with an acronym ending with ‘xx’ include individuals that belong to different color varieties or that were sampled from different regions, even though they belonged to the same breed. They either were kept by different breeders with unknown exchange of genetic material or were sampled in different regions within a country. Therefore, the definition of a population in our study refers to the sampling population rather than a breeding population because this does not apply to some of the populations. For all fancy breeds sampled in Germany, breed names follow the European Poultry Standards [18]. The breed named “Italiener” (Italian), with different color varieties, is a Leghorn type breed for which a separate breed standard exists in Germany.

**Table 1:** The SYNBREED chicken diversity consortium

Contact	Sampling region	Institution
Olivier Hanotte	Albania	School of Life Sciences, University of Nottingham, United Kingdom
Miika Tapio/Mervi Honkatukia	Finland	Luke Natural Resources Institute, Finland
Steffen Weigend	Germany	Friedrich-Loeffler-Institut, Germany
Henner Simianer	Germany	Georg-August-Universität, Germany
András Hidas	Hungary	Institute for Small Animal Research, Hungary
Amadeu Francesch	Spain	IRTA-Centre Mas de Bover, Spain
Christine Flury	Switzerland	School of Agricultural Forest and Food Sciences, Bern University of Applied Sciences, Switzerland
Asmaa Abushady	Egypt	Genetics Department, Faculty of Agriculture, Ain Shams University, Cairo, Egypt.
Olivier Hanotte/Takele Desta	Ethiopia	School of Life Sciences, University of Nottingham, United Kingdom
Ahmad Ali	Pakistan	Department of Bioscience COMSATS, University Islamabad, Pakistan
Mohyeldein Berima	Sudan	Department of Animal Production, Faculty of Agriculture, University of Zalingei, Sudan
Charles Lyimo	Tanzania	Sokoine University of Agriculture, Tanzania
Farai Muchadeyi	Zimbabwe	Agricultural Research Council-Biotechnology Platform, South Africa
Raed M. Al-Atiyat/Riyadh S. Aljumaah	Saudi Arabia	King Saud University, Kingdom of Saudi Arabia
Mohammad Shamsul Alam Bhuiyan	Bangladesh	Department of Animal Breeding and Genetics, Bangladesh Agricultural University, Bangladesh
Guohong Chen	China	Yangzhou University, Jiangsu Province, People’s Republic of China
Mehmet Ali Yildiz	Turkey	Animal Science, Faculty of Agriculture, Ankara University, Turkey
Cuc, Ngo Thi Kim	Vietnam	National Institute of Animal Science, Vietnam
Jeremy Austin / Michael Herrera	Pacific/Philippines	School of Biological Sciences, University of Adelaide, Australia
Maria Rosa Lanari	Argentina	National Institute of Agricultural Technology, Argentina
Fernando Mujica	Chile	Universidad Austral de Chile, Chile
Carl Schmidt	Rwanda/Uganda	University of Delaware, Delaware, USA

Samples from Iceland,Norway, Poland, Russia, Ukraine, France, Italy, Israel, Thailand were taken from the AVIANDIV project (https://aviandiv.tzv.fal.de/, EC project BIO4CT980342)

The populations were classified into twelve categories based on their continent of origin and/or type as shown in Table 2 and Additional file 1: Table S1. In the case of populations of Asian and European origin collected in Germany, the sampling location was also included in the category name (as “DE”). The category Asia_local included native chicken breeds sampled in Asia. Likewise, the category Europe_local comprises breeds of European background sampled in different parts of Europe. The DE_Europe_Ban and DE_Asia_Ban categories consist of bantam type chickens from European and Asian origin, respectively, which were both sampled in Germany. Some of the breeds have already been characterized in other studies (references provided in the last column of Additional file 1: Table S1) mainly using microsatellites.

**Table 2:** Categories of chicken breeds

Category	Full name	Number of breeds	Number of individuals
Wild	Wild type chicken	2	38
Com_WL	Commercial white layers	4	80
Com_BL	Commercial brown layers	4	80
Com_BRO	Commercial broilers	4	73
DE_Europe_Ban	European bantams sampled in Germany	8	156
DE_Europe	European breeds sampled in Germany	35	660
DE_Asia_Ban	Asian bantams sampled in Germany	8	177
DE_Asia	Asian breeds sampled in Germany	28	531
Europe_local	European local breeds sampled across Europe	25	443
Asia_local	Asian local breeds sampled across Asia	30	509
South_America	South American breeds	4	78
Africa	African breeds	22	410
Overall		174	3235

#### Genotyping

DNA samples were genotyped with the Affymetrix® Axiom™ Genome-Wide Chicken Genotyping Array encompassing over 580 K SNPs [27]. Genotyping was performed at the Technische Universität München (Prof. R. Fries). In a few cases, which are marked with an asterisk in Table S1, genotype data was provided by partners.

### Data editing and filtering

In total, genotype information for 580,961 SNPs was obtained from the array. 579,621 of the SNPs were annotated using the genome assembly Gallus_gallus-5.0 [28]. We deleted 134 duplicated SNPs (both SNPs deleted). We only considered SNPs from the 28 autosomal chromosomes and removed 26,839 SNPs from the two sex chromosomes. Furthermore, we deleted 499 SNPs with ambiguous chromosome annotation. We filtered the data for an animal call rate of ≥95% and SNP call rate of ≥99% (leaving 436,581 SNPs) using the SNP & Variation Suite (SVS) version 8.1 [29]. We then performed LD based pruning which has been found to effectively reduce the effects of ascertainment bias in diversity analysis when using SNP data [30]. LD based pruning of SNPs was performed using SVS with the parameters “50 5 0.2”, which represent window size, window shift and r^2^ (pruning of markers with a pairwise r^2^ of greater than 0.2), respectively, leaving 123,273 SNPs for further analysis. Furthermore, imputation was performed on the remaining SNPs using Beagle 3.3 [31] to recover missing genotypes.

### Data analysis

#### Genetic diversity between populations and assessing the population structure

##### Genetic distances and phylogenetic tree

We estimated Reynolds’ genetic distances [32] between the sampled populations. These distances were used to construct an unweighted neighbor joining (NJ) tree using SplitsTree software (version 4.14.4) [33]. Based on the tree we identified possible clusters and labeled them accordingly.

##### Principal component analysis

We performed a principal components analysis (PCA) using SVS. Because of the large number of 3235 individuals, we calculated the average principal component (PC) scores for each population to make their positions in the PCA plot presentable. We then plotted the average scores of each population for PC 1 & 2 with different colors to highlight the breeds’ categories.

##### Admixture analysis

We evaluated the relatedness of the populations through admixture analysis using ADMIXTURE 1.3 software [34]. ADMIXTURE determines population relatedness and assigns populations to ancestral clusters. It includes a cross-validation procedure that allows the identification of a number of populations K which fits best the model based upon cross-validation (CV) error. We analyzed our data set up to a value of K = 80, however without reaching a minimum of the CV error (data not shown). We display results for K = 2 to 11 according to the number of clusters identified with the NJ tree, to illustrate population relatedness and assignment of populations to clusters with proportions to ancestral populations.

#### Genetic diversity within populations

Genetic variability measures such as proportion of polymorphic SNP loci, levels of observed (*H*_*o*_) and expected (*H*_*e*_) heterozygosity were used to evaluate the genetic diversity within populations. The observed heterozygosity was calculated directly, while the expected heterozygosity was estimated as: *H*_*e*_ = 2*q*(1 − *q*) where *q* was the frequency of one of the alleles [35]. The *H*_*e*_ and *H*_*o*_ estimates for all individuals within each population were averaged over all SNPs. Because of low sample sizes and the fact that a number of the populations did not form mating groups, the calculated expected heterozygosity values should be treated with caution. Consequently, for many of the breeds we avoided making conclusions based on the Hardy–Weinberg principles.

## Results

### Genetic diversity between populations and the population structure

#### Neighbor joining tree and cluster assignment

**Fig. 1:**
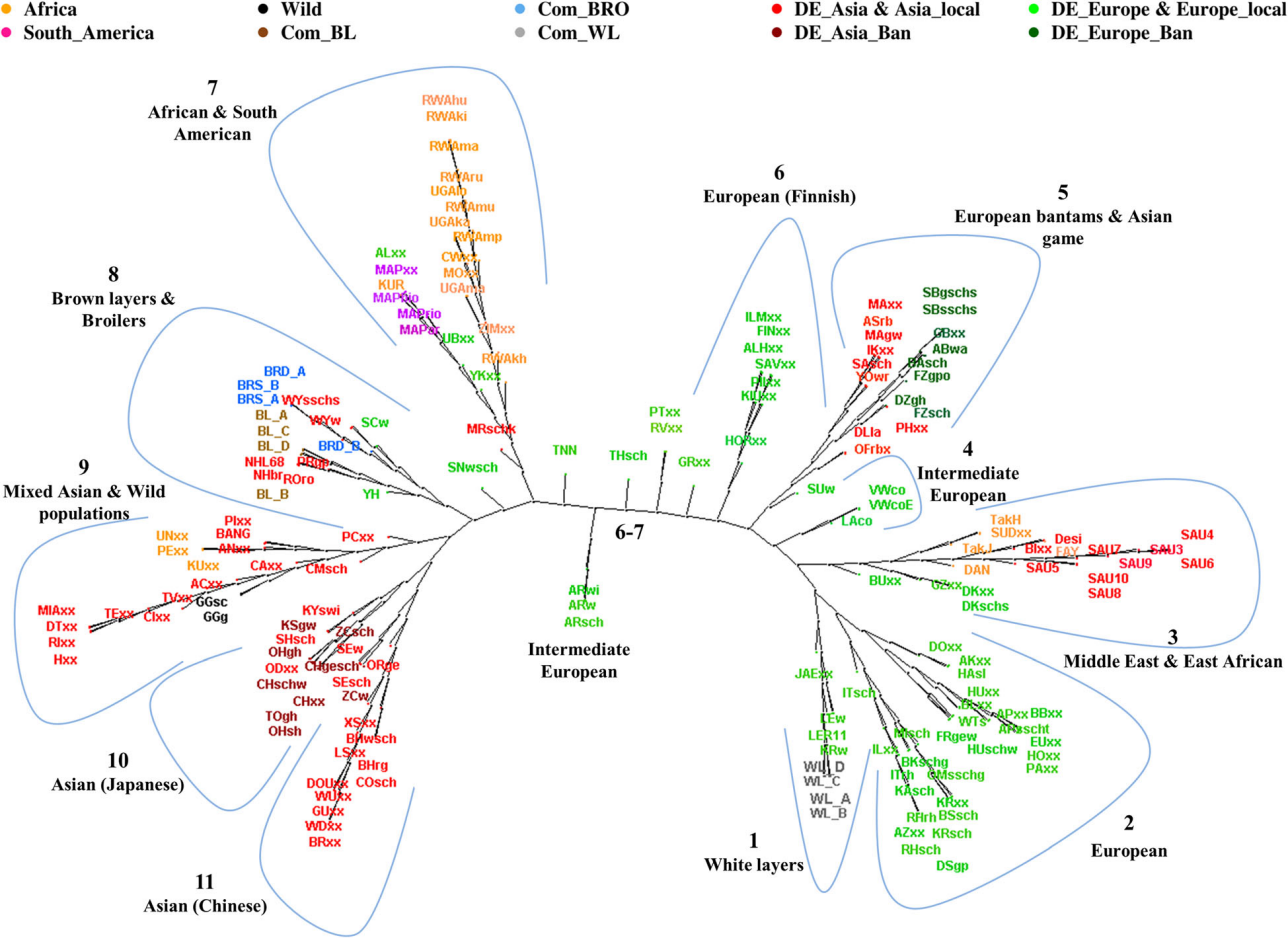
Neighbor Joining tree of 174 chicken populations based on Reynolds’ genetic distances calculated from SNP genotypes. Clusters 1 to 11 are described in the main text and in detail in Additional file 2: Document S1.

The Reynolds’ genetic distances between populations were used to construct a neighbor joining tree which is presented in Fig. 1. We labeled observed clusters on the tree. It should be noted that these clusters were identified manually according to our visual interpretation. Below we provide a general description of the clustering results. More detailed information about the clusters and breeds within each cluster is presented in Additional file 2: Document S1.

In cluster 1, the White Leghorn lines of both commercial and fancy breeds are grouped together. Cluster 2 consists of breeds of European background (green). Cluster 3 encompasses mainly breeds from the Middle East and geographically nearby areas, sampled in Saudi Arabia, Egypt, Pakistan, Israel, Sudan, Ethiopia, Turkey, and Italy. The close relationship of the breeds in this cluster is likely due to their neighboring geographic distribution and distribution routes of chickens in these regions. The NJ tree further shows a very small cluster (cluster 4) which consists of two populations of Vorwerkhuhn (VWco and VWcoE) and Lakenfelder (LAco). Vorwerkhuhn was recognized as a standardized breed in Germany in 1919 and one of the founder breeds was the Lakenfelder breed. Cluster 5 consists of European bantam breeds as well as some Asian game birds which were sampled in Germany. Cluster 6 consists exclusively of chickens sampled in Finland. Following this group, several populations of European background were arranged in the middle of the tree, but were not forming a visually distinct cluster. They were found between cluster 6 and 7. Among these breeds there were three populations of the Araucanas. Cluster 7 branches into two sub-clusters with African populations on the one side and South American Mapuche populations on the other. Among the African populations, there were two Tanzanian ecotypes (MOxx and CWxx). Though five ecotypes from Tanzania were included in this study, they did not cluster together in the NJ tree. The remaining three breeds from Tanzania clustered with populations in cluster 9. The second sub-cluster including the South American sub-cluster also contained some populations from Eastern Europe (Russia, Ukraine and Albania).

In cluster 8 commercial brown layers and broilers are found. Close to the four commercial purebred brown layer lines (BL_A-D), there were also two lines of New Hampshire (NHL68 and NHbr) and the fancy breed of Rhode Island Red (ROro). Two of the brown layer lines (BL_A and BL_B) originated from the breed Rhode Island Red while the other two lines (BL_C and BL_D) are based on White Plymouth Rock. New Hampshires may have formed a part of the dam lines used in the development of brown layer lines [17]. The Plymouth Rocks (PRgp) sampled from fancy breeders clustered close to the purebred broiler lines (BRS_A, BRS_B, BRD_A and BRD_B). Plymouth Rock was part of the female lines for the development of broiler chickens [12]. Even though modern broiler lines became very different from these main founders, it is interesting to see that they clustered together with the fancy breed of Plymouth Rock. Cluster 9 is dominated by breeds of Asian background, mainly from Vietnam. They clustered with three of the Tanzanian ecotypes. Notably, in this cluster the two wild populations (GGg and GGsc) sampled in Thailand were also found. Both clusters 10 and 11 consist of breeds of exclusively Asian background. The breeds in cluster 10 are mainly Japanese and were sampled in Germany. Cluster 11 is dominated by Chinese breeds sampled in both Europe (Germany) and Asia. All the Asian bantam breeds which were sampled in Germany were also found in clusters 10 and 11.

#### Principal component analysis

**Fig. 2:**
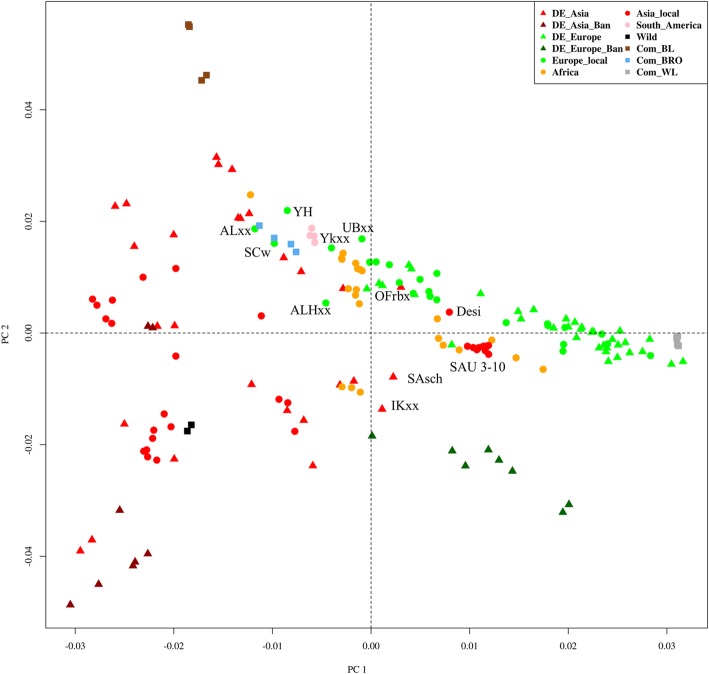
Principal component analysis with components averaged across populations. Breeds which are labelled, their names are mentioned in the main text

Average scores of each population for PC1 versus PC2 are shown in Fig. 2. Populations sampled in Germany are denoted by triangle symbols. The commercial breeds and the two wild populations are displayed as squares, while the rest of the populations are marked as dots. The first PC shows a gradually increasing separation of the European type breeds (green) on the one side from the Asian breeds (red) on the other side, with the African (orange) and South American (pink) breeds in the middle. The Asian breeds sampled in Germany clustered with chicken breeds sampled in Asia. The Mapuche chickens sampled in South America clustered mostly towards the Asians side of the PCA plot, while the African types were separated, with some of them clustering towards the Asian and others towards the European breeds. The Asian breeds (populations also seen in NJ cluster 3) from the Middle East and nearby regions (i.e. Saudi Arabians, Bedouin from Israel, and Desi from Pakistan), Indian game breed (IKxx), Sumatra black (SAsch) and Orloff (OFrbx) clustered with the European breeds and some of the African breeds. A few breeds of (mostly eastern) European origin found in cluster 7 and 8 of the NJ tree included breeds such as the Hungarian Yellows (YH), the Albanian Crowers (ALxx), the Ukrainian bearded (UBxx), the Yurlov crower (YKxx) from Russia, as well as ALH (ALHxx) from Finland and Swiss chicken (SCw). They clustered in the Asian side of the PCA plot with broilers, South American and some of the African breeds. PC1 also shows a wide separation between the two layer line types, the commercial brown layers (in brown colour) on the one end and the white (gray) egg layers on the other. Commercial broilers (light blue) are between them, but much closer to the brown layers.

It is noteworthy that the second PC could be related to the breed’s body size. This is because the PC2 shows a transitioning of mainly the small sized (mostly bantams) birds at the lower part of the PCA plot and the normal sized birds towards the upper part. However, the separation is much clearer for the European type breeds than for the Asian types.

#### Admixture analysis

**Fig. 3:**
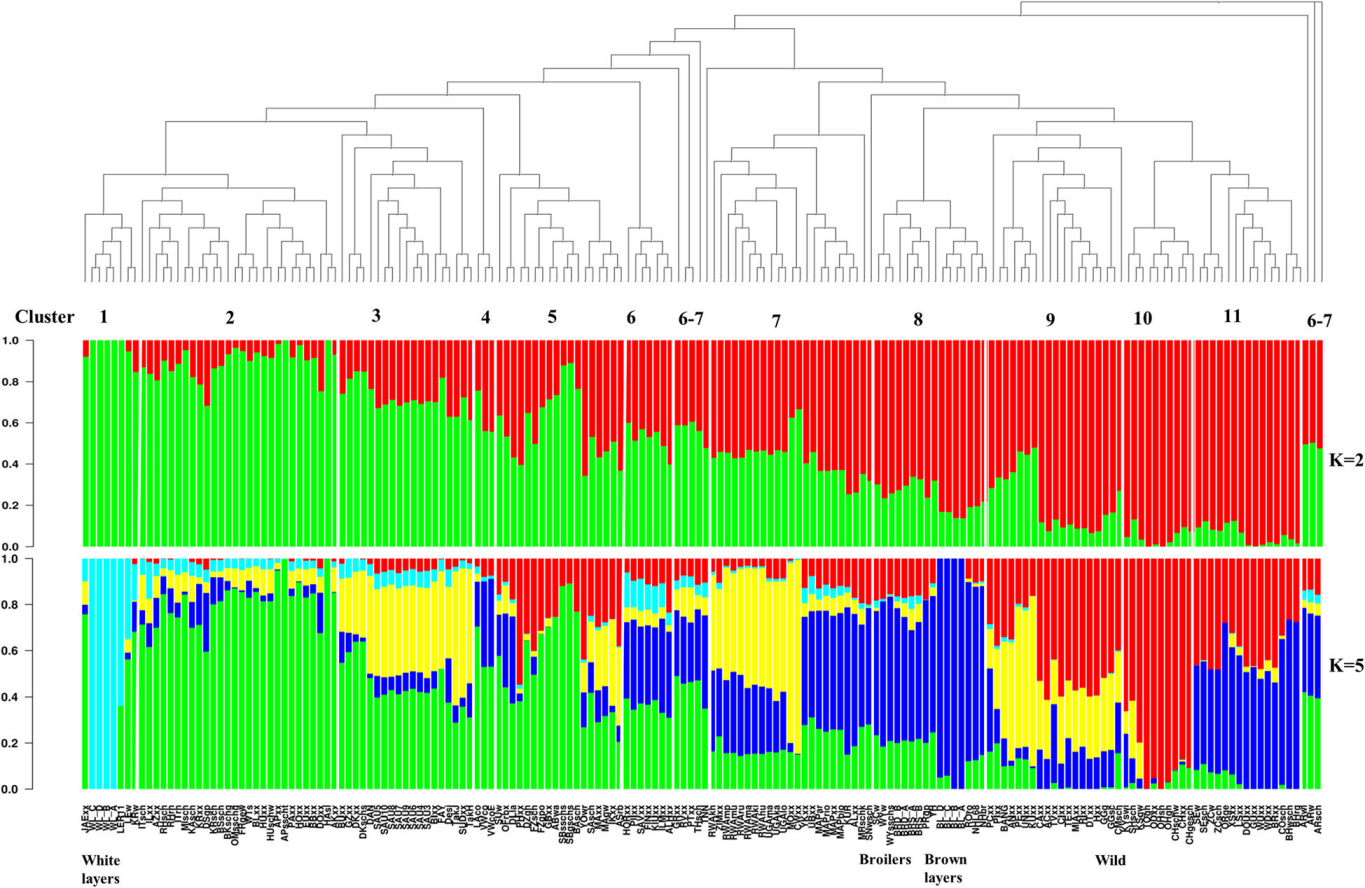
Neighbor Joining tree and admixture analysis of the 174 chicken populations. At the bottom of the NJ tree the cluster numbers are given. Different clusters are separated by white vertical lines in the admixture plots. On the right side of the plots, the assumed numbers of ancestors (K values) used in the admixture analysis are given

Admixture analysis results for K = 2 and K = 5 are displayed in Fig. 3; results for the other K-values up to K = 11 are shown in Additional file 3: Figure S1. We only included in the main text the results for K = 2 to show the overall structure of the studied populations, K = 5 to show the extent of admixture in these populations because K = 5 was visually clear and less dense than K values greater than 5. We transformed the NJ tree from Fig. 1 into a cladogram in order to relate the tree to the admixture plots. Since the Araucana populations are found in the center of the NJ tree in Fig. 1, we used one of them, the Araucana black (ARsch), as the first breed in the cladogram. We then adopted the order of the breeds obtained from the cladogram (clusters 1 to 11 as in Fig. 1) as the order of the breeds in the admixture plots (Fig. 3).

In agreement with the PCA results, the admixture exhibited a gradually increasing separation of breeds from European (green) background from breeds of Asian (red) background with K = 2, with the African and South American breeds situated in the middle of the spectrum. The commercial white layers were completely homogeneous in the European gene pool (green) at K = 2 while the brown layers and broilers were admixed, however, with more proportion of the Asian ancestry cluster. In the NJ tree, cluster 3 is made up of populations from Asia (Saudi Arabia, Pakistan, and Israel) and Africa (Sudan, Egypt and Ethiopia) clustering in the middle of European clusters. On the admixture plot (Fig. 3) these populations of cluster 3 display a larger genome share with Europeans (green). Regarding the African populations, the populations found in NJ clusters 7 and 9 had more affiliation to the Asian gene pool except for the Tanzanian ecotypes Morogoro and Ching’wekwe in NJ cluster 7. The admixture analysis shows that the Morogoro and Ching’wekwe share more European lineage similarly to the African breeds of cluster 3 rather than those of cluster 7. The assignment of these two breeds to cluster 7 on the NJ tree could be due to the one-dimensional nature on the phylogenetic tree with limited capability to resolve the membership when breeds are more related to several other breeds. The South American Mapuche chickens were more affiliated with the Asians at K = 2 as in the PCA plot.

At K = 5, the white layers displayed their own homogenous cluster (light blue) which is not shared among many breeds. Thereby, the White Leghorn line (LER11) was more affiliated to this white layers’ cluster, while the other populations which clustered with the commercial white layer populations in NJ cluster 1 (Fig. 1) were admixed, with more contribution from the European gene pool (green). Two of the brown layer lines (BL_A and BL_B), the two purebred lines based on Rhode Island Red, were also homogeneous (in the blue gene pool) while the other two brown layer lines showed very little admixture with the European gene pool (green). The blue gene pool also dominated in the broiler lines, the South American (Mapuche) and Chinese breeds (NJ cluster 11). It should be noted that the breeds which showed high affiliation to this blue gene pool were located on the upper left box of the PCA plot (see Fig. 2), which is another illustration of their relationship. The yellow gene pool is shared among all the NJ clusters that contained African breeds (clusters 3, 7 and 9). In those NJ clusters one also finds the Middle Eastern populations, a few European and Asian breeds including the two wild populations which also have reasonable affiliation to this gene pool. The Asian breeds in NJ cluster 10 were very little admixed. They were all sampled in Germany and probably have been kept in small flocks with inbreeding taking place. Among these breeds (NJ cluster 10) the Ohiki and Totenko (OHsh and TOgh) were completely homogeneous.

Overall, the populations with Asian background had a higher degree of admixture (with exception of those sampled in Germany) than those of European background which constitute a large proportion of the European (green) cluster. This suggests a higher diversity within the Asian breeds than within the European breeds.

### Genetic diversity within populations

**Fig. 4:**
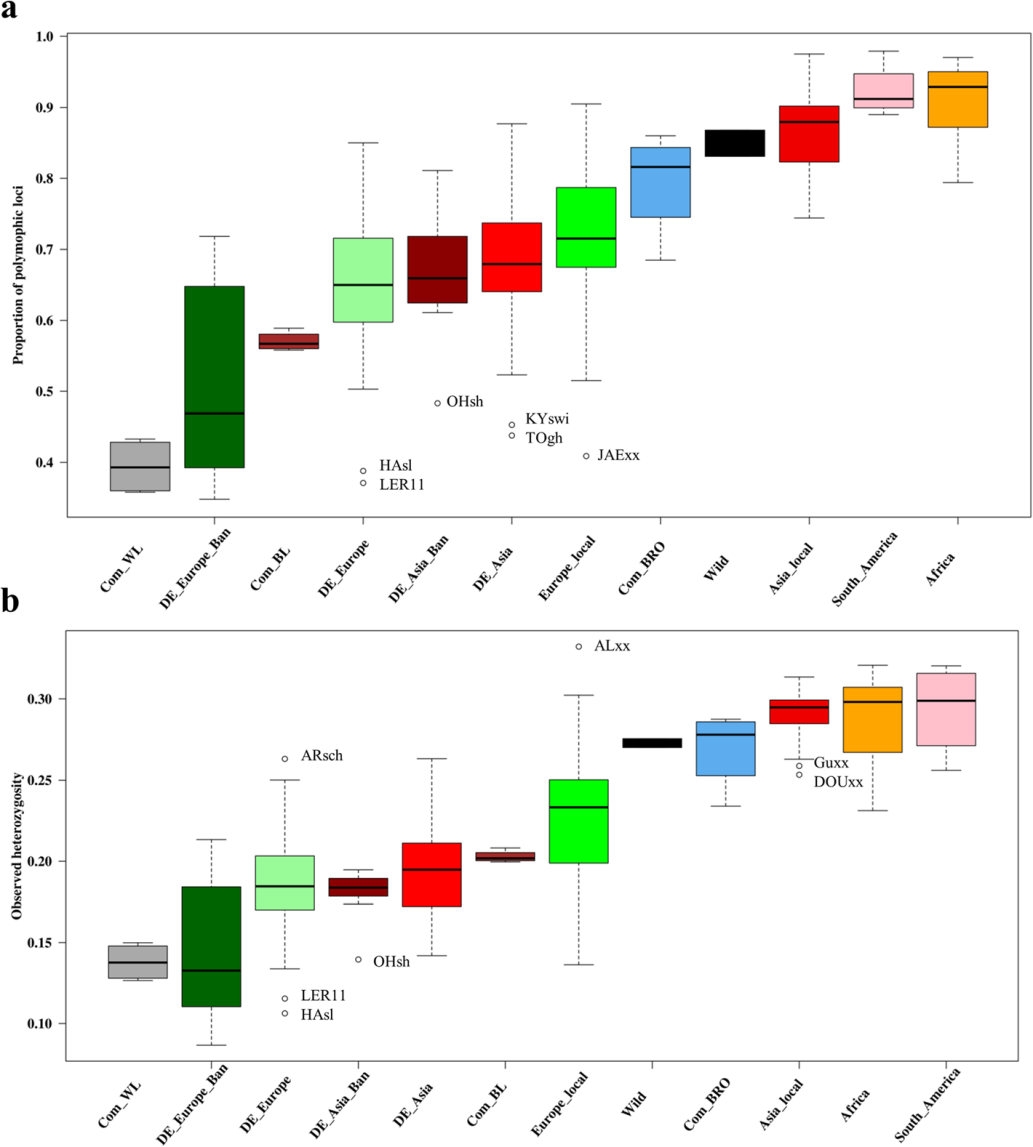
Proportion of polymorphic loci (**a**) and observed heterozygosity (**b**) within the populations grouped by chicken category. ALxx - Albanian Crowers, ARsch - Rumpless Araucana black, DOxx - Dou (Henan game), GUxx - Gushi chicken, HAsl - Hamburgh silver spangled, JAExx - Jaerhoens, KYswi - Koeyoshi Longcrower, LER11 - White Leghorn,OHsh - Ohiki bantam, silver duckwing, TOgh - Toutenko black breasted red

In Fig. 4 we show the proportion of polymorphic loci and mean observed heterozygosity of populations within each category. The proportion of polymorphic loci (*p*), observed (*H*_*o*_) and expected (*H*_*e*_) heterozygosity for each population are shown in Table S1.

The proportion of polymorphic loci was lowest in commercial white layers with $\overline{p\ }$ (average *p* within category) = 0.394 (Figure 4a), followed by the European bantam breeds sampled in Germany (DE_Europe_Ban) with $\overline{p}$ = 0.511 and commercial brown layers with $\overline{p}$ = 0.570. However, the proportion of polymorphic loci varied considerably within the European bantams. Among the commercial lines, the broilers had the highest degree of SNP polymorphism with $\overline{p}$ = 0.794. However, one broiler line (BRD_B) showed a rather low polymorphism, *p* = 0.685, compared to the other three broiler lines with *p* > 0.800. The European breeds sampled in Germany had on average a lower proportion of polymorphic SNP loci ($\overline{p}=$ 0.511) than those from other parts of Europe ($\overline{p}=$ 0.724), which are labelled as “Europe_local”. Within these two European categories, there were three extreme outliers with a very low average proportion (*p* < 0.410) of polymorphic loci, i.e. the Leghorn line (LER11), and the Hamburger silver spangled (HAsl) and the Jaerhoens (JAExx) breeds.

Asian breeds sampled in Germany also had lower proportions of polymorphic loci ($\overline{p}$ = 0.662 and 0.679 for DE_Asia_Ban and DE_Asia, respectively) than those sampled in Asia ($\overline{p}=$ 0.863). Among the Asian bantams sampled in Germany, the Ohiki silver Duckwing (OHsh) breed had an extremely low mean proportion of polymophic loci (*p*= 0.483, Table S1) while the remaining populations of this group displayed average values above 0.600. The Totenko black breasted red (TOgh) and Koeyoshi (KYswi), both breeds of Japanese origin, were outliers among the Asian breeds sampled in Germany with a very low proportion of polymorphic loci (*p* = 0.438 and 0.453 respectively, Table S1). They formed a homogeneous cluster in the admixture analysis plot (part of NJ cluster 10, Figure 3) which may be due to reduced diversity. The breeds sampled in Asia (Asian_local), the South American and the African breeds showed high variability of SNPs, which was even higher than that of the wild populations on average. The wild populations had a $\overline{p}$ of 0.849 while the South Americans and the Africans had 0.923 and 0.912, respectively.

The ${\overline{H}}_o$ over all populations combined was 0.232. Similar to the variation in SNP polymorphism, the level of heterozygosity was very low and deviated more from the overall mean (≤ 0.150) in white layers, some European breeds and European bantam breeds sampled in Germany. The white layers had an ${\overline{H}}_o$ of 0.138. The commercial brown layers had moderate levels of heterozygosity (ranging from 0.200 to 0.208), while the broilers were the most heterozygous among the commercial lines with estimates ranging from 0.234 to 0.287.

The European breeds which were sampled in various parts of Europe other than Germany (Europe_local) had a higher proportion of heterozygous SNPs (with ${\overline{H}}_o$ = 0.228, which is very close to the overall mean heterozygosity of all the studied populations) than the European breeds sampled in Germany (with ${\overline{H}}_o$ = 0.185). Two of the European bantams, the gold and silver Sebright (SBgschs and SBsschs), had the lowest level of heterozygosity among all the breeds. The Sebrights are reported to be highly inbred with small population sizes according to the Central Documentation on Animal Genetic Resources in Germany [36], which goes along with the high degree of homozygosity found.

The Asian breeds sampled in Germany exhibited lower heterozygosity (${\overline{H}}_o$ = 0.196) than those sampled in Asia (${\overline{H}}_o$ = 0.289). The lowest proportion of heterozygous SNPs among the Asian populations was observed for Ohiki silver Duckwing (bantam), Totenko black breasted red and Koeyoshi (which were sampled in Germany), which are also low in the proportion of polymorphic SNPs. Both the African and the Mapuche populations from South America had very high levels of heterozygosity, with an ${\overline{H}}_o$ of 0.288 and 0.294, respectively, while for the two wild populations (GGsc and GGg) the proportion of heterozygous SNPs was slightly lower (${\overline{H}}_o$ = 0.273).

## Discussion

The SYNBREED chicken diversity panel encompasses a global set of chicken breeds. This extensive collection of genetic variability, combined with a high-resolution characterisation of the genome allows deep insights into the diversity within the species, and makes the panel a valuable resource for research. In this study, we focused our analyses on the assessment of genetic relationships between populations to evaluate the distribution of diversity at a global scale, as far as this is represented by the present collection. In addition, we studied the degree of variability within population and compared it between the various categories of breeds. We compared the results of various analyses of the diversity spectrum with our expectations, which were based on sampling sites, historical records, known results from earlier studies and personal knowledge of the breeds’ history.

### Genetic clustering of populations

The various approaches used to assess genetic relationship between chicken populations of the spectrum consistently identified a gradual separation of genomic diversity from Asian to European breeds, with populations from Africa and South America located towards the center. This becomes evident in the Admixture analysis results, in particular at a resolution level of K = 2 clusters, as well as in the plot of the first two PCs, but also in assessing the origin of cluster members in the phylogenetic tree. The majority of Asian breeds sampled either in Asia (China, Vietnam, Pakistan, Bangladesh, Southeast Asia) or sampled in Germany from fancy breeders grouped together in the NJ tree (clusters 9, 10, and 11) as well as in the PCA plot, but separated from the majority of the European breeds which segregate in NJ clusters 1 and 2. The wild populations fitted well into the Asian cluster. They display high levels of genetic diversity as shown by the levels of heterozygosity, SNP polymorphism and their high admixture. This finding is in agreement with the widely accepted opinion that chickens were first domesticated in Asia, predominantly from the Red Jungle Fowl (*Gallus gallus*), with some contribution from *Gallus sonneratii* in Southwest India [37] and probably *Gallus lafayettii* in Sri Lanka (reviewed by [3, 10]), and then spread to various continents. Another general observation confirming earlier studies based upon microsatellites is that commercial white layer and brown layer breeds clustered separately at opposite ends of the diversity spectrum [25, 38]. Together with broiler lines they cover only a limited part of the spectrum and a wide diversity exists complementary to the commercial lines.

#### Chicken of Asian origin

The Asian breeds covered a huge spectrum of genetic diversity. Despite some of the breeds being sampled in Germany (see ‘Methods’ section and Table S1), they blended very well on the PCA plot, NJ tree and admixture analysis with those sampled in Asia. This was also observed in a previous study based on microsatellite markers [19]. It shows that the breeds of Asian background that are kept by fancy breeders in Germany, even though some of them have been kept for over 150 years, they still belong to the Asian gene pool. They are mostly kept as purebreds to maintain their specific phenotypic features which are of interest to fancy breeders. For example, the Cemani (CMsch) breed which was sampled in Germany has its roots from Indonesia. A typical phenotypic trait of the breed is dermal hyperpigmentation (fibromelanosis), a mutation which makes the chicken entirely black [39]. The Indonesian local type of this breed is closely related to the Green and Red Jungle Fowls due to continuous interbreeding of the breed with the Jungle Fowls and other domestic chickens [40]. Likewise, in our study it is clustered closely to the Red Jungle Fowls in NJ cluster 9 so they didn’t lose such relatedness. On the other note, the fanciers chose to keep the Asian ornamental breeds for their miniature features (i.e. Ohiki, Chabo and Ko Shamo) and long crowing and/or fighting features (i.e. Totenkou, Koeyoshi, Shamo and Onaga dori) and their ornamental long tail traits as well [44]. So these breeds remained closely related to the local Asian breeds. Another notable observation is that in cluster 3 of the NJ tree, some of the Asian breeds sampled in the Middle East clustered with African and European breeds. This is also supported by the PCA plot as well as the admixture analysis plot. The close relationship of these breeds could be supported by their geographic distribution, though it is not clear whether this resulted from migration of chickens from Asia to Africa along the Indian Ocean, and from Europe and the Arabian Peninsula via the Mediterranean and the Red Sea [44, 45], or from a continuous exchange of the Mediterranean region with that part of Asia.

#### Chickens of European origin

The European breeds sampled in Germany clustered very well with the rest of the European breeds. Consistent with that, breeds of European origin are represented close together in the PCA plot, distinct from the breeds of Asian origin (PC1). The second PC distinguishes bantam breeds from large chicken breeds in the European gene pool. The Iron Age is assumed to be the main period for dispersion of chickens through Europe. Our results suggest that the majority of breeds categorized as typical European breeds according to the European Poultry Standards (those categorized as DE_Europe and DE_Europe_Ban) have been little or not exposed to crossbreeding with Asian breeds as they do not overlap with Asian breeds. However, there are some exceptions for local breeds sampled in Europe. In the PCA plot (Figure 2), a few breeds, mainly from Eastern Europe (Russia, Hungary, Albania, and Ukraine), but also from Switzerland and Finland are found away from other European breeds and clustered more towards the Asian breeds. As mentioned above, one of the routes for chickens from Asia to Europe was through Russia and Eastern Europe. Given the history of separation between the East and the West of Europe, the affiliation of the Eastern European breeds (found in clusters 6–8) to the Asian breeds might suggest that they have been bred rather isolated from other European (Western and Northwestern) breeds, and therefore have not yet lost their relatedness to breeds of their origin even after being in Europe for a long time. Subsequently breeds such as the Finnish lines (in cluster 6), Hungarian and Polish Green legged Partridge (GRxx) chickens have been kept in conservation flocks after the reunification of the East and the West [38, 41, 42]. Finland has been part of the East under the Russian Empire until 1917. Further information on these Finnish, Hungarian and Polish chickens can be found in Additional file 2. Alternatively, some of the European breeds clustering in the neigbourhood of Asian breeds might have been exposed to crossbreeding with Asian type breeds as is documented for the Swiss chicken (Schweizer Huhn) (http://www.fao.org/dad-is/browse-by-country-and-species/en/). Indeed the Swiss chicken, Transylvanian Naked Neck and Hungarian Yellow do show slightly higher levels of observed heterozygosity than expected which may suggest possible crossbreeding.

#### Chickens of African and South American origins

Chickens in Africa and South America originated from both Asian and European chickens [6, 9]. None of the African and South American populations appeared at any extreme points, neither in the NJ tree nor in the PCA plot but were in the middle either slightly towards an Asian or a European affiliation. However, South American populations were underrepresented in this panel which is not representing a complete picture of South American chicken diversity, while African populations were better represented and therefore can potentially cover a reasonable spectrum of the African diversity.

##### African

The split of African breeds between both Asian and European clusters supports the reports on their origin from both an Asian and a European origin [8]. Mitochondrial DNA studies have also shown that the common haplogroup in the African chickens is shared with some Asian and European chickens [3, 53] while other haplogroups observed in Africa (less common and possibly of more recent arrival) included those also observed in commercial layers and broilers as well as in Northwest Europe [21]. Consistant with that, some of the African breeds are clustered not far from the commercial broilers in the PCA plot, while on the admixture plot (K = 5) they have a good share of the gene pool (blue) which segregate in the commercial brown layers, broilers and Chinese populations. We believe this relationship is possibly due to the fact that they share some similar ancestries tracing back from Asia. The studied African populations were sampled in the North, East and South of Africa. The North (from Egypt and Sudan) and the East (from Ethiopia, Horn of Africa) African breeds were grouped closely together with the Saudi Arabian breeds and share a high proportion of the European gene pool (Figure 3). The relationship between these breeds is explained above. The breeds from Uganda, Rwanda, Tanzania (partly), and Zimbabwean ecotypes were clustered together. The distribution of the African breeds suggests that there might be some gene flow between them as they were sampled in geographically close countries. The splitting of the Tanzanian breeds into two groups (clusters 7 and 9) supports the two maternal origins reported previously [43]. The Ugandan chickens were clustered together in the same sub-cluster of NJ cluster 7. The Kuroiler breed, however, also sampled in Uganda, did not cluster very close with the other Ugandan breeds. It is reported that Kuroiler chickens were derived by crossbreeding either colored broiler males with Rhode Island Red females, or White Leghorn males with female Rhode Island Reds [44]. They have recently been brought from India to Uganda through a project which aimed at improving sustainability and productivity (meat and eggs) of chickens in Africa. In this line, African populations also shared very little of the genome with Kuroilers as displayed in the admixture plot (Figure 3; the yellow part prevailing in African populations is almost missing in Kuroilers). Instead, there was a high degree of overlap of the Kuroiler genome with breeds in cluster 8 where brown layers and broilers dominated, as well as a shared ancestry with cluster 11 of Chinese breeds.

##### South American

South American breeds were exclusively represented by the Mapuche chickens in this study. These populations showed a good share of affiliation with the Chinese populations at K = 5 (Figure 3) of the admixture analysis, but also with some membership into the European (green) lineage. In the PCA plot they seemed slightly more related to Asian breeds (Figure 2), while they can be found in a rather central position between European and Asian breeds in the NJ tree (Cluster 7). Even though a previous study of the eggshell coloration in Chilean breeds suggested a possible Chinese origin [45], Wang et al. [46] and Wragg et al. [47] later reported that the blue egg shell trait in the Chilean and Chinese breeds has a different genetic basis in the two origins. It was reported that many phenotypic features of the Mapuche chickens resemble those of breeds of Asian origin rather than of European origin [48], but these populations showed a level of admixture with both the Asian and European gene pool in our analysis. Our current results do not really solve the debate regarding the origin of the Mapuche chickens. Another point of interest is that the Mapuche did not cluster with the European Araucanas, but still the admixture plot shows a lot of overlap between them. In fact, all the gene pools segregating in Mapuche chicken also segregate in the Araucanas for all the K values; the only difference is the proportion of affiliations to the gene pools. For example, the Araucanas at K = 9 - 11 show higher proportions of the lineages segregating in European populations (green and gray) than the Mapuche. Therefore, it is possible that the European Araucanas might have been mixed with the European breeds, or some of their genomes are getting fixed rapidly as they do show lower levels of observed heterozygosity than expected and their genetic diversity is highly reduced compared to that of the Mapuche.

### The distinction of within breed diversity between local and fancy breeds

The highest genetic diversity was observed within populations sampled in Africa, South America and Asia, some of which exhibited even higher diversity than the wild populations. Generally, the local type breeds from the four continents exhibit more genetic diversity than those from German fancy breeders and commercial layer lines (Figure 4). The local chickens are often kept by villagers under extensive management systems and without controlled breeding programs, but in some cases they are also kept in conservation facilities with the purpose to preserve their genetic architecture [20, 42, 49, 50]. Therefore, the high genetic diversity persists due to the fact that the pool for mating individual is generally larger, and hence a lower rate of inbreeding, and there is some exchange of genetic material by intercrossing of breeds, and little artificial selection is practiced [21, 40, 51]. Although the chicken breeds kept by German fancy breeders cover a wide spectrum of diversity overlapping with local breeds, the management followed by the fancy breeders only preserved the genetic relatedness of these breeds to their ancestral genetic background which, however, caused a drastic reduction in the level of genetic diversity within the breeds. This is likely due to several reasons: Firslty, for the fancy breeds that were imported from Asia to Europe, the number of animals was limited. Therefore, this founder effect contributed to a reduced level of diversity compared to the original populations. Secondly, fancy breeds in Germany (of both Asian and European background) are generally kept in small flock sizes with little or no exchange of mating stocks between breeders. Taking the example of Cemani breed again, the local Cemani breed in Indonesia has shown a similar level of nucleotide diversity as the Jungle Fowls [40]. However, in our study, the Cemani from fancy breeders, even though it was brought only recently to Germany, already has reduced genetic diversity compared to the *Gallus gallus* species and also had the lowest genetic diversity among the local Asian populations of its respective NJ cluster 9. This is probably the result of a limited number of breeding individuals and the absence of a continuous gene flow from other breeds while trying to keep the breed pure (as it is for many fancy breeds in Germany) for its interesting fibromelanosis trait. Another examples are the Leghorn line (LER11) and Hamburger breeds which have the lowest genetic diversity within the European categories. The LER11 is a White Leghorn line which has been kept as a closed population at least since 1965 when it came to the Institute for Small Animal Breeding in Celle (Germany) and was most likely based on a narrow gene pool (as other commercial White Leghorn lines). The Hamburger breed is a fancy breed with a small effective population size [52]. Additionally, the selection practices to meet the European breed standards may also have had a huge impact on the reduction of genetic diversity within the fancy breeds. These standards are very strict and breeders aim at an almost “perfect” phenotype. To achieve this, they practice even matings of very close relatives. This is very evident as almost all the Asian and most of the European breeds, which were sampled from fancy breeders, exhibited lower observed heterozygosity within the population than expected (Table S1). A systematic management of diversity in small populations is almost completely missing, and hence all these breeds and color variants display a low level of within breed diversity.

### Genetic diversity of the commercial lines

With respect to commercial purebred lines, white layers are clearly distinct from brown egg layers, while broiler lines cluster more in the center (Figure 2) and closer to Asian than to European breeds. This, in turn, fits well with the history of these chicken lines. Commercial white egg layer lines originated from an Italian breed located in Livorno (Tuscany, central Italy), the single comb White Leghorn. Consequently, they clustered with other European breeds, especially the fancy White Leghorn lines (LER11 and LEW). The genetic basis of brown egg layers is broader than that of white layers, utilizing Rhode Island Red, Plymouth Rock, Australorps, and New Hampshire among others and the broilers were mainly based on Cornish (Indian Game) and Plymouth Rock [12]. The latter might also explain the closer relationship of broilers to brown layers than to white layers as they share some parental background in the White Plymoth Rock. There is a reported loss of ancestral genetic diversity by 50% in commercial lines [17]. In our study, the single-parented white layers have shown much reduced genetic diversity and displayed a homogeneous cluster in the admixture analysis for all K-values that were analyzed. The brown layers had low to moderate genetic diversity. Compared to the layer lines, commercial broilers were more diverse, which is almost at the same level as that of wild populations. This might be related to a broader genetic basis of founder populations and a larger effective population sizes in selection programs.

Measures are needed to preserve genetic diversity, between and within breeds. There is a large spectrum of between breed diversity preserved in the local breeds from different origins and the fancy breeds from Germany. Additionally, the local chickens have proven to be great reservoirs of within breed genetic diversity. However, the low genetic diversity within the fancy breeds, and the non-structured, non-monitored breeding programs of local breeds raised concerns about their vulnerability to go extinct [20, 42, 53]. So measures for preserving and maintaining genetic diversity should include new utilization possibilities of local breeds, but from a genetic point, such breeds should be included in conservation programs. These programs will include both cryopreservation in gene banks and in-situ conservation flocks managed properly to minimize the rate of inbreeding. Flocks should be kept in high numbers to avoid non-random mating and vulnerability to genetic drift effects otherwise smaller number of birds in conversation flocks would results in reduction of genetic diversity over time as it has been observed in some of the already established facilities e.g. [20, 50].

## Conclusions

In this study we assessed genetic diversity between and within breeds from chickens collected across the world, from various backgrounds. It is very evident that the origin, geographic expansion, selection and different management practices have had a major impact on the global pattern of chicken diversity. Overall, the commercial white layers had the lowest variation among the commercial lines, the bantams displayed lower genetic diversity than the normal sized breeds in the respective category of origin (e.g. Asian bantams vs Asian locals or Asians sampled in Germany); and breeds that were sampled in Germany (both European and Asian breeds) had lower genetic diversity than those sampled in various places in the respective continent of origin. At the current state, the commercial breeding lines seem to have not yet reached selection plateau in the current breeding programs and are still responding to the breeders’ objectives. However, the limited diversity they cover (as shown by PCA and NJ tree), and the very low within breed diversity, in particular within the white layer purebred lines, might limit the flexibility to respond to unforeseen future needs. There is still more genetic diversity within the less selected African, South American and some local Asian and European breeds. Therefore, it is required that genetic diversity in these chickens be maintained in order to have the opportunity to respond to future challenges.

As conservation measures are costly, it was stressed by [54] that “conservation decisions must be based on a global inventory of the species diversity”. The data of SCDP can be seen as a step towards establishing such a reference collection in chicken. In this way, it is supporting international initiatives as at the European level with the EU project IMAGE (http://www.imageh2020.eu/index.php), with collaborative effort to characterize and manage genetic diversity in livestock and poultry species. Not only is this panel the biggest gene pool of chicken data by far; it also has the potential to expand as new breeds and other sources of genetic materials will be added from other parts of the world. The SCDP data set presents ample opportunities for exploitation for further chicken molecular genetic studies and is made available for public access (see section “Availability of data and materials” for details).

## Additional files

Additional file 1: **Table S1.** Population information, population diversity measures and origin of DNA samples. (DOCX 88 kb)

Additional file 2: Document S1. Description of clusters. (DOCX 46 kb)

Additional file 3: **Figure S1.** Admixture Analysis. (PDF 366 kb)

## Abbreviations

B.C.: Before Christ
BDRG: Bund Deutscher Rassgeflügelzüchter
CV: Cross validation
DNA: Deoxyribonucleic acid
EDTA: Ethylenediamine tetraacetic acid
EU: European union
GEH: The Society for the conservation of old and endangered livestock breeds
*H*_*e*_: Expected heterozygosity
*H*_*o*_: Observed heterozygosity
LD: Linkage disequilibrium
mtDNA: Mitochondrial DNA
NJ: Neighbor joining
PC: Principal component
PCA: Principal components analysis
SCDP: SYNBREED chicken diversity panel
SNP: Single nucleotide polymorphism
SVS: SNP Variation Suite
SYNBREED: Synergistic plant and animal breeding
