# Supplementary material for: The SYNBREED chicken diversity panel: a global resource to assess chicken diversity at high genomic resolution
Source: BMC Genomics. 2019 May 7;20:345. doi: 10.1186/s12864-019-5727-9 (PMC6505202; doi:10.1186/s12864-019-5727-9)
Supplement: Supplementary file 1 — Table S1. Population information, population diversity measures and origin of DNA samples. (DOCX 88 kb) [file 12864_2019_5727_MOESM1_ESM.docx]

**Table S1 Population information, population diversity measures and origin of DNA samples**

| **Label** | **Local population name^1^** | **English population name^2^** | **No. Ind.** | **Sampling Country** | **NJ Cluster^3^** | $\boldsymbol{H}_{\boldsymbol{e}}$**^4^** | $\boldsymbol{H}_{\boldsymbol{o}}$**^5^** | $\boldsymbol{p}$**^6^** | **Origin of sample^7^** |
| --- | --- | --- | --- | --- | --- | --- | --- | --- | --- |
| **Africa** |  |  |  |  |  |  |  |  |  |
| CWxx | Ching’wekwe |  | 18 | Tanzania | 7 | 0.254 | 0.261 | 0.841 | [1] |
| DAN | Dandarawi |  | 19 | Egypt | 3 | 0.260 | 0.267 | 0.829 | Asmaa Abushady |
| FAY | Fayoumi |  | 17 | Egypt | 3 | 0.253 | 0.259 | 0.794 | Asmaa Abushady |
| KUR | Kuroiler |  | 22 | Uganda | 7 | 0.306 | 0.321 | 0.926 | Asmaa Abushady |
| KUxx | Kuchi |  | 19 | Tanzania | 9 | 0.270 | 0.273 | 0.857 | [1] |
| MOxx | Morogoro Medium |  | 19 | Tanzania | 7 | 0.272 | 0.271 | 0.909 | [1] |
| PExx | Pemba |  | 19 | Tanzania | 9 | 0.303 | 0.311 | 0.935 | [1] |
| RWAhu | Rwanda Huye |  | 25 | Rwanda | 7 | 0.302 | 0.296 | 0.970 | [2] * |
| RWAkh | Rwanda Kirehe |  | 5 | Rwanda | 7 | 0.287 | 0.311 | 0.814 | [2] * |
| RWAki | Rwanda Kicukiro |  | 15 | Rwanda | 7 | 0.300 | 0.299 | 0.939 | [2] * |
| RWAma | Rwanda Matimba |  | 15 | Rwanda | 7 | 0.297 | 0.296 | 0.937 | [2] * |
| RWAmp | Rwanda Mpanga |  | 10 | Rwanda | 7 | 0.295 | 0.306 | 0.899 | [2] * |
| RWAmu | Rwanda Musanze |  | 15 | Rwanda | 7 | 0.308 | 0.307 | 0.950 | [2] * |
| RWAru | Rwanda Rubavu |  | 15 | Rwanda | 7 | 0.303 | 0.307 | 0.939 | [2] * |
| SUDxx | Large Beladi |  | 15 | Sudan | 3 | 0.265 | 0.251 | 0.872 | [3] |
| TakH | Horro |  | 29 | Ethopia | 3 | 0.268 | 0.252 | 0.932 | Olivier Hanotte* |
| TakJ | Jarso |  | 30 | Ethopia | 3 | 0.255 | 0.231 | 0.925 | Olivier Hanotte* |
| UGAka | Uganda Kamuli |  | 21 | Uganda | 7 | 0.304 | 0.299 | 0.966 | [2] * |
| UGAlo | Uganda Lowero |  | 24 | Uganda | 7 | 0.306 | 0.301 | 0.970 | [2] * |
| UGAma | Uganda Masaka |  | 21 | Uganda | 7 | 0.306 | 0.301 | 0.961 | [2] * |
| UNxx^#^ | Unguja |  | 17 | Tanzania | 9 | 0.301 | 0.309 | 0.923 | [1] |
| ZIMxx | Ecotype |  | 20 | Zimbabwe | 7 | 0.313 | 0.297 | 0.967 | [4] |
| **Asia_local** |  |  |  |  |  |  |  |  |  |
| ACxx | Ac |  | 19 | Vietnam | 9 | 0.273 | 0.278 | 0.840 | [5] |
| ANxx | Aseel Pakistan |  | 15 | Pakistan | 9 | 0.288 | 0.297 | 0.877 | Ahmad Ali |
| BANG | Naked Neck |  | 30 | Bangladesh | 9 | 0.309 | 0.301 | 0.944 | Shamsul Bhuiyan |
| BIxx | Bedouin |  | 20 | Israel | 3 | 0.278 | 0.297 | 0.871 | [6] |
| BRxx | Baier chicken |  | 18 | China | 11 | 0.258 | 0.272 | 0.788 | [7] |
| CAxx | Chahua chicken |  | 19 | China | 9 | 0.256 | 0.264 | 0.777 | [7] |
| CIxx | Choi |  | 19 | Vietnam | 9 | 0.279 | 0.302 | 0.839 | [5] |
| Desi | Desi Pakistan |  | 6 | Pakistan | 3 | 0.271 | 0.300 | 0.782 | Ahmad Ali |
| DOUxx | Dou (Henan game) |  | 20 | China | 11 | 0.233 | 0.253 | 0.758 | [8] |
| DTxx | Dong Tao |  | 18 | Vietnam | 9 | 0.277 | 0.269 | 0.847 | [5] |
| GUxx | Gushi chicken |  | 20 | China | 11 | 0.233 | 0.259 | 0.744 | [7] |
| Hxx | Ho |  | 19 | Vietnam | 9 | 0.287 | 0.294 | 0.873 | [5] |
| LSxx | Langshan chicken | Langshan | 20 | China | 11 | 0.251 | 0.304 | 0.772 | [7] |
| MIAxx | Mia |  | 20 | Vietnam | 9 | 0.304 | 0.295 | 0.918 | [5] |
| PCxx | Malakula |  | 4 | Pacific | 9 | 0.279 | 0.291 | 0.757 | Jeremy Austin & Michael James Herrera |
| PIxx | Kibawe Bukidnon |  | 34 | Phillipinen | 9 | 0.318 | 0.298 | 0.975 | Jeremy Austin & Michael James Herrera |
| RIxx | Ri |  | 19 | Vietnam | 9 | 0.307 | 0.298 | 0.925 | [5] |
| SAU10 | Saudi10 |  | 12 | Saudi Arabia | 3 | 0.297 | 0.299 | 0.903 | Raed Al-Atiyat* |
| SAU3 | Saudi3 |  | 11 | Saudi Arabia | 3 | 0.294 | 0.288 | 0.886 | Raed Al-Atiyat* |
| SAU4 | Saudi4 |  | 12 | Saudi Arabia | 3 | 0.296 | 0.299 | 0.902 | Raed Al-Atiyat* |
| SAU5 | Saudi5 |  | 11 | Saudi Arabia | 3 | 0.299 | 0.294 | 0.901 | Raed Al-Atiyat* |
| SAU6 | Saudi6 |  | 12 | Saudi Arabia | 3 | 0.294 | 0.285 | 0.894 | Raed Al-Atiyat* |
| SAU7 | Saudi7 |  | 11 | Saudi Arabia | 3 | 0.296 | 0.291 | 0.893 | Raed Al-Atiyat* |
| SAU8 | Saudi8 |  | 12 | Saudi Arabia | 3 | 0.295 | 0.298 | 0.894 | Raed Al-Atiyat* |
| SAU9 | Saudi9 |  | 11 | Saudi Arabia | 3 | 0.294 | 0.300 | 0.889 | Raed Al-Atiyat* |
| TExx | Te |  | 18 | Vietnam | 9 | 0.295 | 0.300 | 0.882 | [5] |
| TVxx | Tau Vang |  | 19 | Vietnam | 9 | 0.318 | 0.287 | 0.955 | [5] |
| WDxx | Wannan Three yellow |  | 20 | China | 11 | 0.306 | 0.314 | 0.923 | [7] |
| WUxx | Wugu |  | 20 | China | 11 | 0.259 | 0.263 | 0.823 | [8] |
| XSxx | Xiaoshan chicken |  | 20 | China | 11 | 0.280 | 0.291 | 0.854 | [7] |
| **Com_BL** |  |  |  |  |  |  |  |  |  |
| BL_A | Rhodeländer | Rhode Island Red | 20 | commercial | 8 | 0.192 | 0.201 | 0.572 | SYNBREED |
| BL_B | Rhodeländer | Rhode Island Red | 20 | commercial | 8 | 0.190 | 0.202 | 0.558 | SYNBREED |
| BL_C | Weiße Plymouth Rock | White Plymouth Rock | 20 | commercial | 8 | 0.195 | 0.208 | 0.589 | SYNBREED |
| BL_D | Weiße Plymouth Rock | White Plymouth Rock | 20 | commercial | 8 | 0.186 | 0.200 | 0.562 | SYNBREED |
| **Com_BRO** |  |  |  |  |  |  |  |  |  |
| BRD_A | Broiler dam line |  | 17 | commercial | 8 | 0.276 | 0.287 | 0.827 | [6] |
| BRD_B | Broiler dam line |  | 18 | commercial | 8 | 0.226 | 0.234 | 0.685 | [6] |
| BRS_A | Broiler sire line |  | 19 | commercial | 8 | 0.280 | 0.284 | 0.860 | [6] |
| BRS_B | Broiler sire line |  | 19 | commercial | 8 | 0.265 | 0.272 | 0.805 | [6] |
| **Com_WL** |  |  |  |  |  |  |  |  |  |
| WL_A | Weiße Leghorn | White Leghorn | 20 | commercial | 1 | 0.138 | 0.146 | 0.433 | SYNBREED |
| WL_B | Weiße Leghorn | White Leghorn | 20 | commercial | 1 | 0.139 | 0.150 | 0.424 | SYNBREED |
| WL_C | Weiße Leghorn | White Leghorn | 20 | commercial | 1 | 0.120 | 0.127 | 0.358 | SYNBREED |
| WL_D | Weiße Leghorn | White Leghorn | 20 | commercial | 1 | 0.120 | 0.129 | 0.362 | SYNBREED |
| **DE_Asia** |  |  |  |  |  |  |  |  |  |
| ASrb | Asil, rotbunt | Aseel, red mottled | 20 | Germany | 5 | 0.236 | 0.208 | 0.810 | SYNBREED |
| BHrg | Brahma, rebhuhnfarbig | Brahma, partridge | 20 | Germany | 11 | 0.212 | 0.192 | 0.662 | SYNBREED |
| BHwsch | Brahma, weiß-schwarzcolumbia | Brahma, white-black columbia | 20 | Germany | 11 | 0.212 | 0.194 | 0.688 | SYNBREED |
| CMsch | Cemani, schwarz | Cemani,black | 13 | Germany | 9 | 0.251 | 0.241 | 0.755 | SYNBREED |
| COsch | Cochin, schwarz | Cochin, black | 21 | Germany | 11 | 0.249 | 0.240 | 0.768 | SYNBREED |
| DLla | Deutsche Lachshühner, lachsfarbig | German Faverolles salmon | 20 | Germany | 5 | 0.214 | 0.207 | 0.704 | SYNBREED |
| IKxx | Indische Kämpfer, verschiedenen Farben | Indian Game, various colors | 20 | Germany | 5 | 0.195 | 0.174 | 0.638 | SYNBREED |
| KYswi | Koeyoshi | Koeyoshi Longcrower | 11 | Germany | 10 | 0.148 | 0.154 | 0.453 | SYNBREED |
| MAgw | Malaien,gold-weizenfarbig | Malay, gold-wheaten | 20 | Germany | 5 | 0.230 | 0.211 | 0.746 | SYNBREED |
| MAxx | Malaien, verschiedene Farben | Malay, various colors | 10 | Germany | 5 | 0.233 | 0.230 | 0.697 | SYNBREED |
| MRschk | Marans, schwarz-kupfer | Marans, black-copper | 20 | Germany | 7 | 0.277 | 0.263 | 0.877 | SYNBREED |
| NHbr | New Hampshire, gold- braun | New Hampshire, red brown | 20 | Germany | 8 | 0.223 | 0.220 | 0.718 | SYNBREED |
| NHL68 | New Hampshire, gold-braun | New Hampshire, red brown | 19 | Germany | 8 | 0.222 | 0.211 | 0.650 | SYNBREED |
| ODxx | Onaga dori, verschiedene Farben | Onagadori, various colors | 14 | Germany | 10 | 0.165 | 0.168 | 0.523 | SYNBREED |
| OFrbx | Orloff, rotbunt | Orloff, red colorful | 23 | Germany | 5 | 0.185 | 0.170 | 0.646 | SYNBREED |
| ORge | Orpington, gelb | Buff Orpington | 20 | Germany | 11 | 0.182 | 0.158 | 0.599 | SYNBREED |
| PHxx | Phoenix, verschiedene Farben | Phoenix, various colors | 21 | Germany | 5 | 0.233 | 0.191 | 0.740 | SYNBREED |
| PRgp | Plymouth Rock, gestreift | Barred Plymouth Rock | 20 | Germany | 8 | 0.203 | 0.195 | 0.660 | SYNBREED |
| ROro | Rhodeländer, dunkelrot | Rhode Island Red red | 20 | Germany | 8 | 0.196 | 0.185 | 0.611 | SYNBREED |
| SAsch | Sumatra, schwarz | Sumatran, black | 20 | Germany | 5 | 0.194 | 0.186 | 0.726 | SYNBREED |
| SEsch | Seidenhühner, schwarz | Silkies black | 19 | Germany | 11 | 0.229 | 0.197 | 0.734 | SYNBREED |
| SEw | Seidenhühner, weiß | Silkies white | 20 | Germany | 11 | 0.183 | 0.162 | 0.640 | SYNBREED |
| SHsch | Shamo, schwarz | Shamo, black | 20 | Germany | 10 | 0.266 | 0.252 | 0.833 | SYNBREED |
| SNwsch | Sundheimer, weiß-schwarzcolumbia | Sundheimer, white-black columbia | 20 | Germany | 7_8 | 0.210 | 0.201 | 0.653 | SYNBREED |
| TOgh | Totenko, goldhalsig | Toutenko black breasted red | 21 | Germany | 10 | 0.141 | 0.142 | 0.438 | SYNBREED |
| WYsschs | Wyandotten, silber schwarzgesäumt | Wyandotte, silver-laced | 20 | Germany | 8 | 0.192 | 0.157 | 0.718 | SYNBREED |
| WYw | Wyandotten, weiß | Wyandotte, white | 19 | Germany | 8 | 0.198 | 0.195 | 0.671 | SYNBREED |
| YOwr | Yokohama, weiß-rot gezeichnet | Yokohama, white-red drawn | 20 | Germany | 5 | 0.194 | 0.175 | 0.641 | SYNBREED |
| **DE_Asia_Ban** |  |  |  |  |  |  |  |  |  |
| CHgesch | Chabo, gelb mit schwarzen Schwanz | Japanese Bantam black tailed buff | 22 | Germany | 10 | 0.217 | 0.184 | 0.691 | SYNBREED |
| CHschw | Chabo, schwarz mit weißen Tupfen | Japanese Bantam black mottled | 27 | Germany | 10 | 0.228 | 0.195 | 0.745 | SYNBREED |
| CHxx | Chabo, verschiedene Farben | Japanese bantam, various colors | 29 | Germany | 10 | 0.248 | 0.188 | 0.811 | SYNBREED |
| KSgw | Ko Shamo, gold-weizenfarbig | Ko Shamo, gold-wheaten | 20 | Germany | 10 | 0.187 | 0.183 | 0.638 | SYNBREED |
| OHgh | Ohiki, goldhalsig | Ohiki bantam, red duckwing | 19 | Germany | 10 | 0.177 | 0.174 | 0.611 | SYNBREED |
| OHsh | Ohiki, silberhalsig | Ohiki bantam, silver duckwing | 20 | Germany | 10 | 0.147 | 0.140 | 0.483 | SYNBREED |
| ZCsch | Zwerg Cochin, schwarz | Pekin Bantam,black | 20 | Germany | 11 | 0.204 | 0.183 | 0.660 | SYNBREED |
| ZCw | Zwerg Cochin, weiß | Pekin Bantam,white | 20 | Germany | 11 | 0.198 | 0.191 | 0.659 | SYNBREED |
| **DE_Europe** |  |  |  |  |  |  |  |  |  |
| AKxx | Altenglische Kämpfer, verschiedene Farben | Carlise Old English Game, various colors | 18 | Germany | 2 | 0.182 | 0.177 | 0.584 | SYNBREED |
| APsscht | Appenzeller Spitzhaube, silber-schwarz getupft | Appenzeller Pointed Hood silver spangled | 18 | Germany | 2 | 0.146 | 0.134 | 0.503 | SYNBREED |
| ARsch | Araucanas, schwarz | Rumpless Araucana black | 20 | Germany | 6_7 | 0.267 | 0.263 | 0.850 | SYNBREED |
| ARw | Araucanas, weiß | Rumpless Araucana white | 7 | Germany | 6_7 | 0.248 | 0.220 | 0.716 | SYNBREED |
| ARwi | Araucanas, wildfarbig | Rumpless Araucana black breasted red | 20 | Germany | 6_7 | 0.237 | 0.218 | 0.740 | SYNBREED |
| BBxx | Brabanter, verschiedene Farben | Brabanter, various colors | 9 | Germany | 2 | 0.195 | 0.193 | 0.580 | SYNBREED |
| BKschg | Bergische Kräher, schwarz-goldbraungedobbelt | Bergische Crower, black-goldenbrown | 31 | Germany | 2 | 0.196 | 0.179 | 0.702 | SYNBREED |
| BLxx | Brakel, verschiedene Farben | Brakel, various colors | 20 | Germany | 2 | 0.187 | 0.151 | 0.592 | SYNBREED |
| BSsch | Bergische Schlotterkämme, schwarz | Bergische Schlotterkaemme, black | 20 | Germany | 2 | 0.225 | 0.197 | 0.742 | SYNBREED |
| DOxx | Dorking, verschiedene Farben | Dorking, various colors | 20 | Germany | 2 | 0.234 | 0.203 | 0.789 | SYNBREED |
| DSgp | Deutsche Sperber | German Grey Chickens cuckoo | 19 | Germany | 2 | 0.232 | 0.210 | 0.806 | SYNBREED |
| EUxx | Eulenbarthühner, verschiedene Farben | Owlbeard Chicken, various colors | 12 | Germany | 2 | 0.211 | 0.206 | 0.650 | SYNBREED |
| FRgew | Friesenhuhn, gelb-weißgeflockt | Frisian Fowl chamois pencilled | 20 | Germany | 2 | 0.195 | 0.163 | 0.626 | SYNBREED |
| HAsl | Hamburger, silberlack | Hamburgh silver spangled | 19 | Germany | 2 | 0.106 | 0.106 | 0.388 | SYNBREED |
| HOxx | Holländer Weißhauben, verschiedene Farben | Poland White Crested, various colors | 17 | Germany | 2 | 0.186 | 0.168 | 0.613 | SYNBREED |
| HUschw | Houdan, schwarz-weißgescheckt | Houdan, black mottled | 8 | Germany | 2 | 0.175 | 0.181 | 0.517 | SYNBREED |
| ITrh | Italiener, rebhuhnhalsig | Leghorn brown | 20 | Germany | 2 | 0.203 | 0.181 | 0.656 | SYNBREED |
| ITsch | Italiener, schwarz | Leghorn black | 19 | Germany | 2 | 0.195 | 0.182 | 0.659 | SYNBREED |
| KAsch | Kastilianer, schwarz | Castilians black | 19 | Germany | 2 | 0.209 | 0.203 | 0.697 | SYNBREED |
| KRsch | Krüper, schwarz | Creeper black | 29 | Germany | 2 | 0.240 | 0.210 | 0.807 | SYNBREED |
| KRw | Krüper, weiß | Creeper white | 30 | Germany | 1 | 0.210 | 0.207 | 0.709 | SYNBREED |
| KRxx | Krüper, verschiedene Farben | Creeper, various colors | 14 | Germany | 2 | 0.212 | 0.200 | 0.679 | SYNBREED |
| LAco | Lakenfelder | Lakenfelder | 20 | Germany | 4 | 0.202 | 0.167 | 0.674 | SYNBREED |
| LER11 | White Leghorn | White Leghorn | 20 | Germany | 1 | 0.124 | 0.115 | 0.371 | SYNBREED |
| LEw | White Leghorn | White Leghorn | 20 | Germany | 1 | 0.192 | 0.163 | 0.605 | SYNBREED |
| MIsch | Minorka, schwarz | Black Minorca | 20 | Germany | 2 | 0.183 | 0.172 | 0.603 | SYNBREED |
| OMsschg | Ostfriesische Möwen, silber-schwarzgeflockt | East Friesian Gulls silver pencilled | 20 | Germany | 2 | 0.190 | 0.174 | 0.628 | SYNBREED |
| PAxx | Paduaner, verschiedene Farben | Poland, various colors | 23 | Germany | 2 | 0.227 | 0.172 | 0.725 | SYNBREED |
| RHrh | Rheinländer, rebhuhnhalsig | Rhinelander Chicken, brown | 20 | Germany | 2 | 0.194 | 0.191 | 0.615 | SYNBREED |
| RHsch | Rheinländer, schwarz | Rhinelander Chicken, black | 20 | Germany | 2 | 0.186 | 0.184 | 0.590 | SYNBREED |
| SUw | Sultanhühner, weiß | Sultan, white | 12 | Germany | 5 | 0.196 | 0.190 | 0.603 | SYNBREED |
| THsch | Thüringer Barthühner, schwarz | Thuringian Bearded Chicken, black | 17 | Germany | 6_7 | 0.257 | 0.250 | 0.805 | SYNBREED |
| VWco | Vorwerkhuhn | Vorwerk | 19 | Germany | 4 | 0.214 | 0.198 | 0.715 | SYNBREED |
| VWcoE | Vorwerkhuhn | Vorwerk | 20 | Germany | 4 | 0.190 | 0.196 | 0.609 | SYNBREED |
| WTs | Westfälische Totleger, silber | Westphalian Chicken, silver | 20 | Germany | 2 | 0.164 | 0.143 | 0.550 | SYNBREED |
| **DE_Europe_Ban** |  |  |  |  |  |  |  |  |  |
| ABwa | Antwerpener Bartzwerge, wachtelfarbig | Barbue d'Anvers quail | 20 | Germany | 5 | 0.146 | 0.141 | 0.492 | SYNBREED |
| BAsch | Bantam, schwarz | Bantam, black | 20 | Germany | 5 | 0.130 | 0.124 | 0.446 | SYNBREED |
| DZgh | Deutsche Zwerghühner, goldhalsig | German Bantam, gold partridge | 20 | Germany | 5 | 0.208 | 0.176 | 0.661 | SYNBREED |
| FZgpo | Federfüßige Zwerghühner, gold-porzellanfarbig | Booted Bantam, millefleur | 20 | Germany | 5 | 0.130 | 0.125 | 0.424 | SYNBREED |
| FZsch | Federfüßige Zwerghühner, schwarz | Booted Bantam,black | 19 | Germany | 5 | 0.196 | 0.193 | 0.635 | SYNBREED |
| GBxx | Grübbe Bartzwerge, verschiedene Farben | Barbue du Grubbe,various colors | 18 | Germany | 5 | 0.229 | 0.213 | 0.718 | SYNBREED |
| SBgschs | Sebright, gold | Sebright Bantam, gold | 19 | Germany | 5 | 0.107 | 0.097 | 0.361 | SYNBREED |
| SBsschs | Sebright, silber | Sebright Bantam,silver | 20 | Germany | 5 | 0.093 | 0.087 | 0.348 | SYNBREED |
| **Europe_local** |  |  |  |  |  |  |  |  |  |
| ALHxx | ALH |  | 15 | Finland | 6 | 0.220 | 0.200 | 0.731 | Luke^8^, Finland |
| ALxx | Albanian Crowers |  | 19 | Albania | 7 | 0.299 | 0.332 | 0.905 | Olivier Hanotte |
| APxx | Appenzeller Spitzhaube | Appenzeller Pointed Hood | 26 | Switzerland | 2 | 0.197 | 0.156 | 0.675 | Christine Flury |
| AZxx | Appenzeller Barthuhn | Appenzeller | 14 | Switzerland | 2 | 0.222 | 0.221 | 0.691 | Christine Flury |
| BUxx | Sicilian Buttercup |  | 20 | Italy | 3 | 0.200 | 0.193 | 0.666 | [6] |
| DKschs | Denizlikräher, schwarz-silber | Denizli Long Crowers, black-silver | 11 | Germany | 3 | 0.162 | 0.182 | 0.515 | SYNBREED |
| DKxx | Denizli | Denizli Long Crowers | 20 | Turkey | 3 | 0.231 | 0.222 | 0.739 | Mehmet Ali Yildiz |
| FINxx | TYR |  | 15 | Finland | 6 | 0.292 | 0.220 | 0.888 | Luke^8^, Finland |
| GRxx | Green legged Partridge |  | 20 | Poland | 6_7 | 0.226 | 0.233 | 0.705 | [6] |
| GZxx | Gerze | Gerze, black | 20 | Turkey | 3 | 0.187 | 0.187 | 0.622 | Mehmet Ali Yildiz |
| HORxx | HOR |  | 12 | Finland | 6 | 0.181 | 0.177 | 0.656 | Luke^8^, Finland |
| HUxx | Houdan |  | 20 | France | 2 | 0.216 | 0.220 | 0.664 | AVIANDIV |
| ILMxx | ILM |  | 13 | Finland | 6 | 0.236 | 0.241 | 0.703 | Luke^8^, Finland |
| ILxx | Icelandic Landrace |  | 20 | Iceland | 2 | 0.261 | 0.238 | 0.812 | [6] |
| JAExx | Jaerhoens |  | 20 | Norway | 1 | 0.134 | 0.136 | 0.409 | [6] |
| KIUxx | KIU |  | 15 | Finland | 6 | 0.264 | 0.254 | 0.806 | Luke^8^, Finland |
| PIIxx | PII |  | 15 | Finland | 6 | 0.220 | 0.199 | 0.709 | Luke^8^, Finland |
| PTxx | Prathuhn | Catalana chicken | 20 | Spain | 6_7 | 0.233 | 0.251 | 0.730 | Francesch, Amadeu |
| RVxx | Red Villafranquina |  | 20 | Spain | 6_7 | 0.241 | 0.250 | 0.715 | AVIANDIV |
| SAVxx | SAV |  | 10 | Finland | 6 | 0.264 | 0.248 | 0.787 | Luke^8^, Finland |
| SCw | Schweizer Huhn | Swiss chicken | 19 | Switzerland | 8 | 0.252 | 0.256 | 0.757 | Christine Flury |
| TNN | Transilvanien Naked Neck | Transylvanian Naked Neck | 20 | Hungary | 6_7 | 0.237 | 0.244 | 0.743 | [9] |
| UBxx | Ukrainian bearded |  | 19 | Ukraine | 7 | 0.288 | 0.302 | 0.881 | AVIANDIV |
| YH | Hungarian Yellow |  | 20 | Hungary | 8 | 0.233 | 0.241 | 0.712 | [9] |
| YKxx | Yurlov Crower |  | 20 | Russia | 7 | 0.278 | 0.295 | 0.869 | [6] |
| **South_America** |  |  |  |  |  |  |  |  |  |
| MAPar | Mapuche | Araucana | 19 | Argentina | 7 | 0.275 | 0.256 | 0.915 | Maria Rosa Lanari |
| MAPbio | Mapuche | Araucana | 35 | Chile | 7 | 0.316 | 0.311 | 0.979 | Fernando Mujica |
| MAPrio | Mapuche | Araucana | 14 | Chile | 7 | 0.291 | 0.287 | 0.890 | Fernando Mujica |
| MAPxx | Mapuche | Auracana | 10 | Chile | 7 | 0.307 | 0.320 | 0.909 | Fernando Mujica |
| **Wild** |  |  |  |  |  |  |  |  |  |
| GGg | Gallus Gallus Gallus |  | 20 | Thailand | 9 | 0.285 | 0.275 | 0.868 | [6] |
| GGsc | Gallus Gallus Spadiceus |  | 18 | Thailand | 9 | 0.277 | 0.270 | 0.831 | [6] |

Populations are grouped in different categories. German breeds are divided into categories according to the German Standard of Perfection indicating their supposed origin. Remaining populations are classified according to their sampling area

1 – Name of the chicken population in the sampling region (German names according to the German Standard of Perfection)

2 – English name of the chicken population (for German breed names partially based on https://wikivisually.com/wiki/List_of_German_chicken_breeds)

3 – Numbers indicate the affiliation of populations to the clusters identified from the Neighbor Joining tree presented in Figure 1

4 – Estimates of expected heterozygosity per population

5 – Estimates of observed heterozygosity per population

6 – Proportion of polymorphic loci per population

7 – DNA samples were collected within the SYNBREED project, taken from previous studies (references), sampled in a previous collaborative EC project AVIANDIV (BIO4CT980342) or provided by partners (partner names) of the SYNBREED Chicken Diversity Consortium. Names marked “*” indicate that partners provided SNP genotype data

8 - Luke Natural Resources Institute, Finland

**References**

1. Lyimo CM, Weigend A, Msoffe PL, Eding H, Simianer H, Weigend S. Global diversity and genetic contributions of chicken populations from African, Asian and European regions. Anim Genet. 2014;45:836–48.

2. Fleming DS, Koltes JE, Markey AD, Schmidt CJ, Ashwell CM, Rothschild MF, et al. Genomic analysis of Ugandan and Rwandan chicken ecotypes using a 600 k genotyping array. BMC Genomics. 2016;17.

3. Berima M eldein A, Yousif IA, Eding H, Weigend S, Musa HH. Population structure and genetic diversity of Sudanese native chickens. African J Biotechnol. 2013;12:6424–31.

4. Muchadeyi FC, Eding H, Wollny CBA, Groeneveld E, Makuza SM, Shamseldin R. Absence of population substructuring in Zimbabwe chicken ecotypes inferred using microsatellite analysis. 2007;38:332–9.

5. Cuc NTK, Simianer H, Eding H, Tieu H V., Cuong VC, Wollny CBA, et al. Assessing genetic diversity of Vietnamese local chicken breeds using microsatellites. Anim Genet. 2010;41:545–7.

6. Hillel J, Groenen MAM, Tixier-Boichard M, Korol AB, David L, Kirzhner VM, et al. Biodiversity of 52 chicken populations assessed by microsatellite typing of DNA pools. Genet Sel Evol. 2003;35:533–57.

7. Chen G, Bao W, Shu J, Ji C, Wang M, Eding H, et al. Assessment of population structure and genetic diversity of 15 Chinese indigenous chicken breeds using microsatellite markers. Asian-Australasian J Anim Sci. 2008.

8. Granevitze Z, Hillel J, Feldman M, Six A, Eding H, Weigend S. Genetic structure of a wide-spectrum chicken gene pool. Anim Genet. 2009;40:686–93.

9. Bodzsar N, Eding H, Revay T, Hidas A, Weigend S. Genetic diversity of Hungarian indigenous chicken breeds based on microsatellite markers. Anim Genet. 2009;40:516–23.
