# Supplementary material for: The SYNBREED chicken diversity panel: a global resource to assess chicken diversity at high genomic resolution
Source: BMC Genomics. 2019 May 7;20:345. doi: 10.1186/s12864-019-5727-9 (PMC6505202; doi:10.1186/s12864-019-5727-9)
Supplement: Supplementary file 2 — Document S1. Description of clusters. (DOCX 46 kb) [file 12864_2019_5727_MOESM2_ESM.docx]

**Document S1 Description of clusters**

In a Neighbor Joining (NJ) tree constructed from Reynolds’ genetic distance based on SNP data (see Methods in the main text) we identified manually eleven clusters according to our visual observation (Figure 1). Here we provide additional information on the breeds of the SYNBREED Chicken Diversity panel in these clusters. Clusters are visualized in a NJ tree with unweighted branch length which illustrates clustering in a well discernible way.

***Cluster 1*** is dominated by commercial white layers (White Leghorn). WL_A, WL_B, WL_C, and WL_D clustered together with the other White Leghorn lines in the set, LER11 and LEw as well as with the Creeper white (KRw). LER11 is a White Leghorn line maintained at the Institute of Farm Animal Genetics (formerly Institute of Small Animal Breeding in Celle, Germany) since 1965 and has originated from the Cornell Line K [1], while LEw is a German fancy breed of White Leghorns. Interestingly, Jaerhoens (JAExx) from Norway were also found in this cluster. This cluster is closely related with cluster 2 which includes breeds of European background.

***Cluster 2*** splits into two sub-clusters. The first sub-cluster starts with the Italiener black (ITsch) in the middle and then splits into two branches. On one branch there are the two color varieties of the Rheinländer breed, brown (RHrh) and black (RHsch) cluster with Italiener brown (ITrh), Appenzeller Barthuhn (AZxx), which was sampled in Switzerland, and the Icelandic Landrace (ILxx). The second branch of this sub-cluster encompasses German breeds such as Deutsche Sperber (DSgp), Creeper (Krüper KRxx, KRsch) and breeds from the region “Bergisches Land” in Germany (BSsch, BKschg). It is worth noting the close relation of black Creeper (KRsch) to this group, while the white variety of this breed (KRw) clusters with White Leghorn. Furthermore, we find in this group two Spanish breeds, Kastilianer (KAsch) and Minorka (MIsch), which have been bred in Germany for more than 80 years. The second sub-cluster of cluster 2 consists of breeds classified as Northwestern European Breeds (Brakel, BLxx; Westfälische Totleger, WTs; Friesenhuhn, FRgew; and Ostfriesische Möwen, OMsschg) according to the European Poultry Standards [2]. These breeds merge with a cluster of crested breeds, namely Houdan (HUxx, HUschw), Appenzeller Spitzhauben (APsscht, APxx), Brabanter (BBxx), Eulenbarthühner (EUxx), Holländer Weißhauben, (HOxx), and Paduaner (PAxx), and finally merge with the three breeds Hamburger (HAsl), Altenglische Kämpfer (AKxx), and Dorking (DOxx). While Dorking is an old breed brought from England to Germany in the second half of the 19th century, the Hamburger breed belongs to the group of spangled chickens which may have their origin in the Netherlands [3].

***Cluster 3*** encompasses mainly breeds from the Middle East and neighboring areas. It consists of chicken populations sampled in Saudi Arabia (SAU3-10), Egypt (Fayoumi, FAY, and Dandarawi, DAN), Pakistan (Desi), Israel (Bedouin, BIxx), Sudan (SUDxx), and Ethiopia (Horro, TakH, and Jarso, TakJ). At the bottom of the cluster it is complemented by three breeds originating from Turkey, Denizli Kräher (DKschs) sampled in Germany, Denizli (DKxx), and Gerze (GZxx), and the Italian breed Sicilian Buttercup (Buxx).

***Cluster 4*** consists of two populations of Vorwerkhuhn (VWco and VWcoE) and Lakenfelder (LAco). Vorwerkhuhn were bred from 1900 on by Oskar Vorwerk from the breeds Lakenfelder, yellow Orpington, and possibly a few others such as the Andalusian breed and the yellow Ramelsloher.

***Cluster 5*** starts with three populations at the bottom, i.e. Deutsche Lachshühner (DLla), Orloff (OFrbx) and Sultanhühner (SUw) before branching into two sub-clusters. DLla is a meat-type bird originating from the French breed Faverolles. The Orloff, often called Russian Orloff, is considered a Russian breed named after the Russian military and statesman Alexey Grigoryevich Orlov (1737 – 1807) after whom the horse breed ‘Orlov trotters’ is named as well. The Orloffs are believed to have been imported from Persia to Europe in the 17^th^ century and were subsequently promoted by Orlov, but there are speculations that several European breeds including those from Belgium and Germany formed the basis of the breed [4]. The breed has been bred in Germany since the late 19^th^ century and on the PCA plot (Figure 2) it is clustered with populations of European background. Close to the Orloff are the Sultanhühner (SUw), a crested bird which most likely also originates from Eastern Europe (European Poultry Standards).

One sub-cluster of cluster 5 brought together six European bantam breeds (autosomal dwarfism) sampled in Germany. These breeds encompass two color varieties of Sebright (SBgschs and SBsschs), Bantam (BAsch), Antwerpener Bartzwerge (ABwa), Grübbe Bartzwerge (GBxx), two strains of Federfüßige Bartzwerge (FZgpo and FZsch) and Deutsche Zwerghühner (DZgh). DZgh clustered closely with PHxx (Phoenix). This relationship is difficult to explain, but the admixture plot also showed that PHxx is a highly heterogeneous population. The other sub-cluster of this group, which is nearest to the sub-cluster of European Bantam breeds, includes Asian game birds (Malay, MAgw and Maxx; Aseel, ASrb and Indian Game, IKxx) which were sampled in Germany. On the PCA plot (Figure 2), the Malay and Aseel cluster with Asian breeds while the Indian Game cluster is located not far from them but slightly towards the European side of the PCA plot. There are some reports that the Indian Game chickens were actually developed in the United Kingdom in the 1820s and were later accepted for the American breed standards in the 1890s [5]. It is believed that this breed was developed from crosses between Red Aseel and possibly Malay with English game birds [5], which would explain their close relationship in this cluster. This is also supported through their close positioning in the PCA plot. In addition, this sub-cluster is complemented by the breeds Sumatra (SAsch) and Yokohama (YOwr) which were supposedly imported to Germany in the second half of the 19^th^ century from the Sumatra Islands and Japan, respectively.

***Cluster 6*** encompasses European populations sampled in Finland. The genetic diversity of the Finnish breeds (ALHxx, ILMxx, KIUxx, FINxx, SAVxx, PIIxx and HORxx) can to some extent be explained by the breed formation and subsequent management practices. Finnish landraces were developed from several isolated and genetically distinct native populations from remote villages in Finland which survived extintion due to the introduction of commercial hybrids [6]. In order to maintain the genetic and phenotypic diversity of Finnnish Landrace chickens while maintaining the breed’s purity, a conservation programme was formed in 1998 whereby a number of lines of the Landrace are kept. There is also no artificial selection enforced on the Finnish landrace lines except maybe for plumage and legs color [6].

Several populations in the middle of the tree are not forming separate clusters. These breeds include a very diverse set of populations from various countries. Among them are Green legged Partridge (GRxx) from Poland, Spanish breeds Prathuhn (PTxx) and Red Villafranquina (RVxx), Thüringer Barthühner (THsch) from Germany, Transylvanian Naked Neck (TNN) from Hungary, and three subpopulations of the Araucana (ARsch, ARw and ARwi) maintained in Germany. It should be noted that the Araucana subpopulations which were sampled in Germany cluster together, but are distant from the Mapuche chickens sampled in Chile and Argentina. The history of the Transylvanian Naked Neck is similar to that of the Hungarian Yellow. They are believed to have been brought to Hungary from Asia in the 9^th^ century, were they were later mixed with Oriental and Mediterranean chickens [7, 8]. They have been kept in conservation institutes for more than 30 years. The Green legged Partridges (GRxx) are traditional rural chickens of Poland. Consistent with previous findings [9], the Green legged Partridge (GRxx) in our study showed a high level of admixture with both European and Asian lineages. Based on the literature, its European background comes from Sicilian Buttercup from Italy and the Asian background from Silkies [9]. This may present some interesting insights and may be the reason why the Green legged Partridge is found in the middle of the NJ tree since the Sicilian Buttercup (BUxx) and Silkies are very distinct and are found at both ends of the NJ tree (cluster 2 and 11, respectively). Following a decline in the population size due to difficulties to adapt to intensive management systems, the breed was maintained as conversation flock since the 1970s [9]. Little is known about the Thüringer Barthühner, although they have been in Germany for at least 200 years [10].

***Cluster 7*** is divided into two sub-clusters. The first one, the African sub-cluster, consisted of populations from Rwanda (RWAhu, RWAki, RWAma, RWAru, RWAmu, RWAmp, and RWAkh), Uganda (UGAlo, UGAka, and UGAma), Zimbabwe (ZIMxx) and two Tanzanian ecotypes (MOxx and CWxx). The other sub-cluster in this cluster consisted of populations sampled in Europe (Albanian Crowers, ALxx; Ukrainian Bearded, UBxx; Yorlov Crowers, YKxx, and Marans, MRschk) and the Mapuche chickens sampled in South America (MAPbio, MAPxx, MAPrio, and MAPar) with one exception, the Kuroiler chickens (KUR).

In the NJ tree, one population sampled in Germany, the Sundheimer (SNwsch), was found between Clusters 7 and 8. The origin of the Sundheimer, an old German meat-type breed (18^th^ century), is not well documented. However, it is likely that local strains from Southern Germany contributed to the breed development as well as game birds from Belgium and France (<https://de.wikipedia.org/wiki/Sundheimer>, Accessed September 21, 2018).

***Cluster 8*** is divided into two branches. One branch consisted of the commercial purebred brown layer lines. In this branch, there was also the Hungarian breed (YH). This is in agreement with earlier studies based on microsatellite analysis [7] where it was shown that this breed makes a separate cluster from other Hungarian chicken populations. In the other branch of this cluster 8 the four purebred broiler lines are assembled together with Plymouth Rocks. Main contributions to the development of broiler breeding came from a variety of hybrids produced on crosses of a heavy game bird, the Cornish (Indian Game) on the male side, and females of the tall and fertile breed Plymouth Rock [11]. Moreover, as the two lines BRS_A and BRS_B reflect broiler sire lines, they are closely related to the two broiler dam lines (BRD_A and BRD_B). This sub-cluster is completed by color varieties of Wyandottes, white (WYw) and silver-laced (WYsschs), and a breed from Switzerland, Schweizerhuhn (SCw). Wyandottes are of American origin and were admitted to the American Standard in 1883 [3]. The founder populations are unknown, but it is likely that Dark Brahma (BHrg, cluster 11) and Dorking contributed [12]. The Schweizerhuhn (SCw, Swiss chicken) originates from Orpingtons (ORge, cluster 11) and Wyandottes, and was bred in Switzerland since 1903. This breed showed a very small proportion of European lineage in the admixture analysis. Crossbreeding of heavy breeds of Asian origin and those from Europe during breed development 100 to 150 years ago might explain the shared ancestries (admixture analysis, Figure 3) of the populations in this cluster with the European breeds and the large and heavy Asian breeds of cluster 11.

***Cluster 9*** is also divided into two sub-clusters. One branching around the middle containing the remaining three breeds from Tanzania namely Pemba (PExx), Unguja (UNxx) and Kuchi (KUxx), together with breeds from Pakistan (Aseel, ANxx), Bangladesh (BANG) and the Philippines (PIxx). This separation of the Tanzanian populations from those in cluster 7 is in agreement with earlier findings based on microsatellites, where Ching‘wekwe clustered with Morogoro-medium in a STRUCTURE analysis, and Unguja clustered together with Pemba while the Kuchi ecotype formed a separate cluster at higher levels of clustering [13]. The separation might be due to different genetic backgrounds following different waves of chickens to Africa. The other sub-cluster was dominated by chicken breeds sampled in Vietnam. In agreement with earlier studies, the four populations Ho (Hxx), Dong Tao (DTxx), Ri (RIxx), and Mia (MIAxx) sampled in the Red river delta in Vietnam clustered together [14]. In addition, the populations Te (TExx) and Choi (CIxx) sampled at the East Coast of Vietnam are found in this cluster, which is complemented by the Tau Vang (TVxx) and Ac (ACxx) breeds from the Mekong delta. Interestingly, in this cluster the two wild populations (GGg and GGsc) sampled in Thailand are placed as well as the Chahua breed (CAxx) from the Yunnan region in China. Based on microsatellites it was shown in an earlier study that CAxx clustered with a *Gallus gallus spadiceus* population sampled in the Yunnan region, but was separated from other Chinese breeds analyzed in this study [15]. The breed Cemani (CMsch) sampled in Germany, and a mixed group of chickens sampled on islands in the Pacific region (PCxx) are situated between the two sub-clusters. Even though CMsch was sampled from birds kept in Germany, it is a rather young breed in Germany originating from Indonesia. It is only little mixed with European breeds which is also supported by the admixture analysis (Figure 3).

***Cluster 10*** consists of breeds of Asian, mainly Japanese, background, which were sampled in Germany. Chabo and Ohiki are bantam breeds originating from Japan. While Chabo was introduced to Germany as early as 1860, Ohiki came to Germany rather recently in 1995 (European Poultry Standards). For both breeds, various colors were sampled, and according to their breed affiliation, these color varieties cluster together (CHschw, CHxx, CHgesch, and OHgh, OHsh). In close neighborhood to Ohiki another breed from Japan can be found, the Onaga dori breed. This long tailed chicken breed was imported to Germany in the second half of the 19^th^ century. The Onaga dori breed clusters together with Totenko (TOgh), a breed also having its roots in Japan and has been kept in Germany since the 19^th^ century. A sub-branch in this cluster includes Shamo (SHsch), KoShamo (KSgw) and Koeyoshi (KYswi), another three Japanese breeds kept by German fancy breeders. The breed Shamo in Germany goes back to imports from Japan in 1953 and belongs to a group of breeds with a general designation as game fowl in Japan. Ko Shamo (KSgw) is a bantam ornamental breed of this group brought from Japan to Germany in 1990. Koeyoshi (Kyswi) is a long crowing chicken which has been bred in Japan since the 18^th^ century. It came to Germany in 1993.

Lastly, ***cluster 11*** in the NJ tree shows several small sub-clusters of mainly Asian (Chinese) origin sampled in both Europe (Germany) and Asia. It starts with two Silkie breeds (SEw and SEsch) and Zwerg-Cochin (ZCw and ZCsch) from China which were sampled in Germany. The German breed name “Zwerg-Cochin” refers to Cochin Bantams as they are also known in the USA and Canada. They came to Germany in the late 19^th^ century. Its older name was Peking-Bantam and it is similar to the Pekin breed, a Bantam chicken that is based in China. There is still considerable debate concerning whether or not Cochin Bantams are Pekins. It is quite interesting to note that this breed clusters with a breed with some very unusual features, the Silkie. Besides their fluffy plumage that feels like silk, Silkies have black skin and bones, blue earlobes, and five toes on each foot. Their origin is not known. Most likely they go back to ancient China, but they might also have their roots in India or on Java [16]. From Asia they came to the West and were first recorded in Europe in the 18th century, while the breed was recognized officially in North America in 1874. Recent crossbreeding between Silkie and Cochin Bantams is rather unlikely given the special phenotypic traits of Silkie. However, admixture analysis (Figure 3) suggests isolated, but shared, ancestry at the genomic level between both breeds.

The second sub-cluster consists of Chinese breeds sampled in China. Chinese breeds encompass light and medium sized layer type and dual purpose breeds like Wannan Three yellow (WDxx), Baier chickens (BRxx), and Gushi chickens (GUxx) from the Gushi county in the Henan province. Henan Dou Ji (DOU) is a game bird from the same region, while Wugu (WUxx) is a silky bird from Taihe county. Langshan (LSxx) and Xiaoshan (XSxx) are heavy-sized chickens used for meat and dual purposes [15]. In addition, in the last sub-cluster of this group there were also breeds sampled in Germany, but clearly showing their Asian background. Brahma sampled in two color varieties (BHwsch and BHrg) is a very heavy breed. It has been used for meat in the USA before broiler breeding started and was most likely developed from birds imported from China. They came to Europe in the mid of the 19^th^ century. Unlike in the USA were Brahmas were bred for utility qualities, in Europe they were kept strickly for ornamental features following the European Poultry Standards and have lost the utility qualities [17]. Cochin (COsch), is a large chicken breed, derived from a heavy feather-legged chicken breed brought from China to Europe and North America in the mid of the 19^th^ century. Although phenotypically different from Brahma, there seems to be a close genetic relationship between the two breeds. Orpington (ORge), another heavy chicken breed that is found in this cluster, is an old breed developed in England in the late 19^th^ century. It appears to be based on crossing Minorcas and Plymouth Rocks, and then Langshans to create a dual purpose breed [18]. Likewise it makes sense that it clustered very closely to the local Chinese breed Langshan.

**References**

1. Cole RK, Hutt FB. Selection and heterosis in Cornell White Leghorns: A review, with special consideration of interstrain hybrids. Anim Breed Abstr. 1973;41:103–18.

2. Rassegeflügel-Standard für Europa in Farbe. Bund Deutscher Rassegeflügelzüchter (ed.), Howa Druck & Satz GmbH, Fürth. ISBN 987-3-9806597-1-0.

3. American Poultry Association. The American standard of perfection. Boston, Massachusetts: American Poultry Association, Inc.; 1910.

4. The Livestock Conservancy. Russian orloff chicken. https://livestockconservancy.org/index.php/heritage/internal/orloff. Accessed 6 Feb 2018.

5. The Livestock Conservancy. Cornish Chicken. http://livestockconservancy.org/index.php/heritage/internal/cornish. Accessed 6 Feb 2018.

6. Fulton JE, Berres ME, Kantanen J, Honkatukia M. MHC-B variability within the Finnish Landrace chicken conservation program. Poult Sci. 2017;96:3026–30.

7. Bodzsar N, Eding H, Revay T, Hidas A, Weigend S. Genetic diversity of Hungarian indigenous chicken breeds based on microsatellite markers. Anim Genet. 2009;40:516–23.

8. Revay T, Bodzsar N, Mobegi VE, Hanotte O, Hidas A. Origin of Hungarian indigenous chicken breeds inferred from mitochondrial DNA D-loop sequences. Anim Genet. 2010;41:548–50.

9. Siwek M, Wragg D, Sławińska A, Malek M, Hanotte O, Mwacharo JM. Insights into the genetic history of Green-legged Partridgelike fowl: MtDNA and genome-wide SNP analysis. Anim Genet. 2013;44:522–32.

10. Benecke N. Der Mensch und seine Haustiere. Stuttgart: Theiss; 1994.

11. Crawford RD. Poultry genetic resources: evolution, diversity, and conservation. In: Poultry Breeding and Genetics. Amster- dam-Oxford-Newyork-Tokyo: Elsevier; 1990. p. 43–60.

12. Petty EL. Wyandottes: The American Breed with an Indian Name and Eurasian Background. http://www.feathersite.com/Poultry/CGP/Wyand/WyandSPPADec01.html. Accessed 11 May 2018.

13. Lyimo CM, Weigend A, Janßen-Tapken U, Msoffe PL, Simianer H, Weigend S. Assessing the genetic diversity of five Tanzanian chicken ecotypes using molecular tools. S Afr J Anim Sci. 2013;43.

14. Cuc NTK, Simianer H, Eding H, Tieu H V., Cuong VC, Wollny CBA, et al. Assessing genetic diversity of Vietnamese local chicken breeds using microsatellites. Anim Genet. 2010;41:545–7.

15. Chen G, Bao W, Shu J, Ji C, Wang M, Eding H, et al. Assessment of population structure and genetic diversity of 15 Chinese indigenous chicken breeds using microsatellite markers. Asian-Australasian J Anim Sci. 2008;21:331–9.

16. Ekarius C. Storey’s Illustrated Guide to Poultry Breeds. Storey Publishing; 2007.

17. Anonymous. Brahma Chickens. https://poultrykeeper.com/chicken-breeds/brahma-chickens/. Accessed 18 Feb 2019.

18. Anonymous. Geschichte der Orpingtonzucht - Sonderverein Deutscher Orpington-Züchter e.V. https://www.sv-orpington.de/geschichte/. Accessed 11 May 2018.
