# Supplementary material for: The SYNBREED chicken diversity panel: a global resource to assess chicken diversity at high genomic resolution
Source: BMC Genomics. 2019 May 7;20:345. doi: 10.1186/s12864-019-5727-9 (PMC6505202; doi:10.1186/s12864-019-5727-9)
Supplement: Supplementary file 3 — Figure S1. Admixture Analysis. (PDF 366 kb) [file 12864_2019_5727_MOESM3_ESM.pdf]

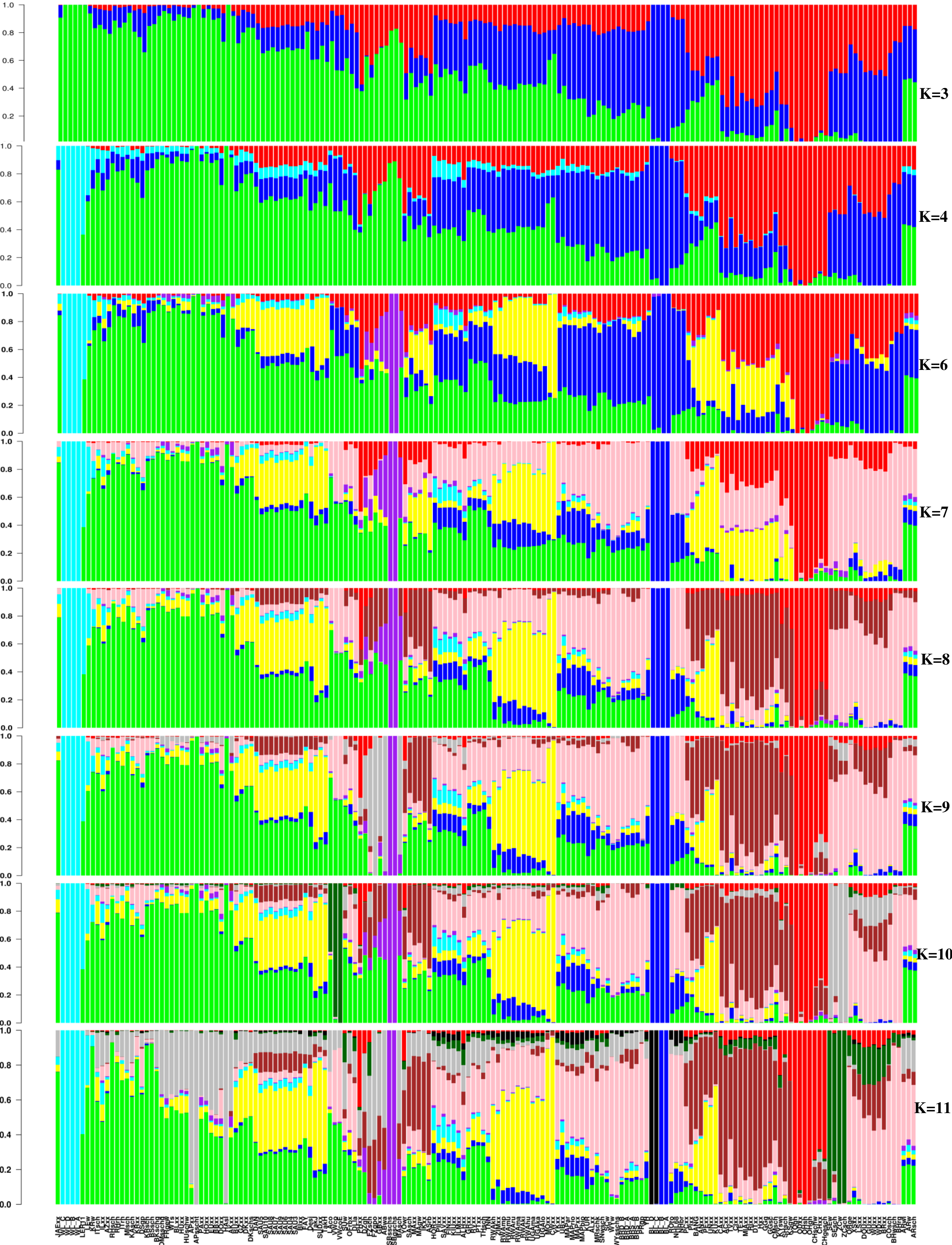

Supplementary figure S1 Admixture Analysis
